# Predicting Emotional Distress, Based on Acquisition, Extinction, Avoidance, and Generalization Learning

**DOI:** 10.1155/2024/6366269

**Published:** 2024-10-23

**Authors:** Naomi Carpentier, Dirk Hermans, Sara Scheveneels

**Affiliations:** ^1^Centre for the Psychology of Learning and Experimental Psychopathology, KU Leuven, Leuven, Belgium; ^2^Clinical Psychology, Maastricht University, Maastricht, Netherlands

## Abstract

This prospective study aimed to investigate whether fear conditioning parameters measured at baseline could predict the development of emotional distress over a 6-month period among 655 first-year university students. Verbal and behavioral measures of acquisition, extinction, avoidance, and generalization were obtained through an online task at the start of the academic year. Emotional distress was evaluated 4 to 6 months later, with questionnaires assessing anxiety, stress, depression, and coping trajectories. Initial analyses explored the interplay of conditioning parameters at baseline, hypothesizing that the corresponding learning processes may mutually reinforce each other, contributing to distinct vulnerabilities for emotional distress. Although no distinct profiles based on conditioning processes were identified, the analyses did uncover correlations between increased acquisition and avoidance of conditioned threat stimuli and reduced extinction, avoidance of safe stimuli, and generalization. Subsequent main analyses related the processes and their interactions to the development of emotional distress. Findings suggest that acquiring fear toward conditioned safety and threat stimuli, as well as avoiding conditioned threat stimuli, may be predictive of higher levels of emotional distress. Analyses relating extinction and generalization to emotional distress revealed mostly nonsignificant findings, emphasizing the need for methodological scrutiny in identifying anxiety-related learning indices. This research contributes to understanding individual differences in the development of emotional distress and informs future investigations into learning processes and their implications for mental health.

## 1. Introduction

In the field of psychopathology, a key question is why certain individuals develop enduring emotional problems following stressful life events while others do not [1–3]. The diathesis-stress model offers a framework for understanding the complex interplay between individual vulnerability factors (i.e., diatheses) and external stressors, such as adverse life events [4–6]. These vulnerability factors, which can be biological or psychological in nature, may predispose individuals to be more susceptible to developing complaints in response to the adverse effects of stress.

Over the past century, learning processes have increasingly come into focus as potential influential diatheses or vulnerability factors, specifically in the development of anxiety problems [7]. Individual differences in learning may determine how individuals cope with stressful events. Understanding learning processes in anxiety can be achieved by building upon a fundamental learning mechanism—fear acquisition.

Fear acquisition involves the development of a fear response (conditioned response; CR) to a previously neutral stimulus (conditioned stimulus; CS+) following repeated pairings with an aversive outcome (unconditioned stimulus; US) [8]. Imagine the experience of failing (US) a set of crucial exams (CS+) despite extensive preparation. For some students, this learning experience may induce a strong emotional response, resulting in a strong fear regarding future exams compared to others. To study individual differences in fear acquisition, researchers often use a laboratory fear conditioning procedure wherein a subject is repeatedly exposed to a neutral stimulus (CS+; e.g., an image) that is immediately followed by an aversive stimulus (US; e.g., electrical stimulation). To control for nonassociative processes impacting the CR, most procedures include a second neutral stimulus (CS−) that is presented alternately with the CS+ and is never paired with the US. Contrary to expectations, cross-sectional evidence indicates that individuals with and without anxiety disorders generally acquire fear responding to the CS+ in a similar way. However, interestingly, the existing evidence further suggests that anxious individuals may struggle more with identifying the CS− as safe [7, 9]. Only a few prospective studies have related individual differences in fear acquisition to the onset of anxiety symptoms. These resulted in mixed findings, both for CS+ responding and CS− discrimination; some studies revealed one or both of these processes to be predictive for the onset of symptoms, while other studies did not [10]. These findings highlight two important points: the necessity for further prospective research to clarify the role of fear acquisition in developing anxiety symptoms and the potential involvement of related processes beyond fear acquisition to the CS+. In this study, we include three related learning processes, which were recently identified as potential contributors to anxiety: impaired extinction, excessive avoidance, and overgeneralization [7].

Impaired fear extinction refers to the persistence of fear despite corrective learning experiences. This can be illustrated by returning to the example of failing exams. After a few subsequent positive experiences of passing other exams, some individuals may quickly regain their trust in their academic abilities. Others might persistently anticipate failure, even when performing well on all subsequent exams (CS is no longer followed by the US). This persistent fear of failure can impact their daily lives, causing extreme stress during study sessions and affecting their overall mental well-being. In a fear extinction procedure in the laboratory, participants are repeatedly exposed to a CS+ without the paired occurrence of a US, which they had previously learned to anticipate [11]. Evidence from cross-sectional studies indicates that patients with anxiety-related disorders show impaired extinction in these laboratory procedures (i.e., higher US expectancies and larger fear responses to the CS+) compared to healthy controls [1]. However, when it comes to the predictive value of individual differences in extinction for the development of anxiety, the evidence is mixed. Some studies found that impaired extinction at baseline could predict the emergence of anxiety following a traumatic event [12–14], while others failed to replicate these findings [15].

Excessive avoidance can be defined as persistent behavior aimed at preventing a negative outcome. Such avoidance behaviors can disrupt daily life and perpetuate anxiety. We can illustrate this again with the impact of failing a set of exams. After their failure, some students may start to persistently avoid academic challenges or opportunities to test their knowledge, resulting in reduced opportunities for academic succes. In laboratory procedures, avoidance is most frequently examined by providing participants with the option to perform a predefined response in the presence of a CS, which effectively prevents the occurrence of the associated US [16, 17]. Recently, researchers have started to include a cost for performing the avoidance response, intending to better identify those individuals who continue to engage in avoidance behaviors despite negatively impacting their daily lives [17, 18]. Cross-sectional studies using these laboratory procedures have found that individuals with anxiety disorders may indeed display more persistent and costly avoidance behaviors compared to healthy controls [19, 20]. However, there is a noticeable lack of prospective studies that examine how individual differences in laboratory avoidance predict future anxiety levels outside of the laboratory context.

Fear generalization occurs when conditioned fear extends to stimuli that are perceptually or conceptually similar to the original CS+. Overgeneralization can significantly magnify the maladaptive consequences of learned fear, causing individuals to react fearfully to a wide range of cues, even to those that do not pose an actual threat [5, 11, 21]. Returning to the example of failing exams, due to excessive fear generalization, individuals may develop anxiety, not only related to exams but also in other academic contexts and evaluative situations. For instance, they might start experiencing anxiety during class discussions or preparing for a driver's test. In the laboratory, researchers have been using a circle-size variation paradigm, first introduced by Lissek et al. [21], to study generalization learning. Here, participants initially associate a specific circle size, either a large or a small circle, with a US during an initial acquisition phase. During the subsequent generalization phase, this circle is presented alongside others of different sizes. This paradigm has been valuable in uncovering differences in generalization patterns between individuals diagnosed with anxiety disorders and healthy controls. Specifically, anxious participants tend to exhibit heightened fear responses to a broader array of generalization cues [22–24]. Prospective research examining the predictive utility of overgeneralization in anxiety, on the other hand, remains very limited. One noteworthy exception is a study by Lenaert et al. [5], which demonstrated that individual differences in generalization during a conditioning task could predict the emergence of anxiety symptoms in university students 6 months later. More precisely, conditioned responding to a generalization stimulus (GS) resembling the CS− more than the CS+ was predictive of heightened anxiety levels.

While these processes have been individually related to anxiety in previous research [1, 5, 12–14, 19, 20, 22–24], how they interact with each other and how these interactions relate to anxiety remains poorly understood [7]. In particular, these learning processes may reinforce each other within individuals, resulting in distinct profiles with varying vulnerability to develop anxiety. For example, following a strongly disappointing exam period (i.e., acquisition), a student with impaired extinction who does not avoid excessively might regain self-confidence after enough subsequent continuous positive academic experiences. Contrastingly, a student displaying strong acquisition, impaired extinction, and excessive avoidance may miss out on these experiences [25], resulting in a higher vulnerability to develop anxiety problems. A student who, in addition, has a tendency to overgeneralize and starts to fear and avoid all kinds of evaluative situations may be even more vulnerable. Based on clinical observations, combinations of the four processes appear to make individuals particularly vulnerable. However, this proposed combined vulnerability has not been investigated so far.

In this exploratory study, we aimed to bridge these gaps by examining the predictive value of laboratory acquisition, extinction, avoidance, and generalization, as well as their interplay, for the development of anxiety. To achieve this, we employed a nonclinical sample, specifically targeting first-year university students. This choice allowed us to identify premorbid markers of anxiety, particularly during a transitional phase characterized by encounters with a broad set of stressors (e.g., academics, finances, social interactions) [5]. Behavioral measures of extinction, avoidance, and generalization in an online task were related to changes in anxiety levels over a 6-month period. In addition to this wider scope, we focused on examining the interplay of these learning processes with stressors, such as failed exams and negative life events (NLEs). Finally, recognizing the high comorbidity between anxiety, stress, and depression [26], we extended our investigation to include stress—and depressive symptoms as outcome measures, seeking to examine whether our learning processes also play a role in their development.

## 2. Method

### 2.1. Participants

A sample of 655 participants took part in the study. These participants were all psychology students at KU Leuven, enrolled in a first-year research participation course. Data collection was spread over three consecutive academic years: 2020–2021, 2021−2022, and 2022–2023. Some minor changes were made to the protocol after the first year of data collection (see Section 2.3).

**Figure 1:**
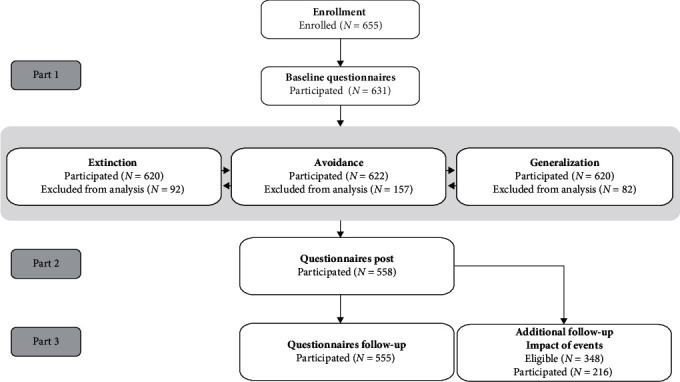
Overview participation rates. *Note*: Part 1 = first three sessions of the study (October); Part 2 = one-session assessment in February; Part 3 = one-session assessment in March.

Two inclusion criteria were applied at sign-up: participants needed to have a sufficient understanding of the Dutch language and could not have signed up for the study in earlier years. Participation in the study was voluntary, and participants were compensated either with course credit or a monetary reward. A number of participants voluntarily withdrew from the study, failed to partake in certain parts, or had to be excluded from analyses on extinction, avoidance, and/or generalization due to inadequate fear acquisition prior to the phase (see Section 2.4). Detailed information about the dropout rates can be found in Figure 1.

The study was conducted in accordance with the ethical guidelines of the KU Leuven Social and Societal Ethics Committee (ethical approval code G-2021-3913).

### 2.2. Materials and Measures

#### 2.2.1. Conditioning Tasks

Conditioning tasks were administered in Part 1 of the study and spread over three consecutive days. Each day commenced with a fear acquisition phase, followed by either the extinction, avoidance, or generalization task.

##### 2.2.1.1. CSs

Two CSs were used in each task: one that signaled threat (CS+) and one that signaled safety (CS−). The conditional stimuli in all tasks were black outlines of geometric shapes presented against a white background. A different set of CSs was used on each day: a triangle and parallelogram on the day of the extinction task, a pentagon and a trapezoid on the day of the avoidance task, and a small and large circle on the day of the generalization task. Which stimulus served as a CS+ and which as a CS− was counterbalanced across participants.

##### 2.2.1.2. USs

USs were selected from the International Affective Picture System (IAPS) [27]. We used nine images previously selected by Lenaert et al. [5], which were categorized into mild, moderate, or severely aversive based on known arousal ratings in young adults [5, 28]. Participants chose between these categories at the beginning of each conditioning task but were encouraged to select the most aversive category, which 69% of participants did. The mild category included images of a firearm, a cockroach, and a paper bag of vomit; the moderate category featured images of tribal mutilation, fecal matter, and a hospitalized infant; and the severe category comprised images of a thoracotomy, a bloodied corpse, and an aggressive dog.

##### 2.2.1.3. US Expectancy and Distress Ratings

Participants rated their expectancy about the occurrence of the aversive image (US) in a 7-s window, immediately concurrent with CS onset. These ratings were provided on a visual analog scale (VAS) ranging from 0 (“certainly no image”) to 10 (“certainly an image”) in one-point steps. Distress was rated in a subsequent 5-s window on a similar VAS ranging from 0 (“no distress at all”) to 10 (“a very large amount of distress”).

#### 2.2.2. Questionnaires

##### 2.2.2.1. Depression Anxiety Stress Scales (DASS-21)

The DASS-21 [29, 30] is a 21-item questionnaire that assesses symptoms of anxiety (DASS-A), stress (DASS-S), and depression (DASS-D) on three subscales. Participants are asked to what degree certain statements applied to them over the past week. The items are rated on a four-point scale, ranging from 0 (“not at all”), over 1 (“to some degree or some of the time”), and 2 (“to a considerable degree or a good part of time”) to 3 (“very much or most of the time”), and result in three subscale sum scores. The subscales showed good reliability in the present study: *α*_DASS-A_ = 0.83; *α*_DASS-S_ = 0.88; *α*_DASS-D_ = 0.90.

##### 2.2.2.2. Generalized Anxiety Disorder Seven Item Scale (GAD-7)

The GAD-7 [31, 32] contains seven items that assess the presence and severity of symptoms of generalized anxiety, which is mostly characterized by excessive worrying. Participants rate symptoms on their occurrence in the past week on a four-point scale ranging from 0 (“not at all”), over 1 (“several days”), and 2 (“more than half the days”), to 3 (“nearly every day”). Responses are summed to obtain a total score. Cronbach's alpha in the present sample was *α* = 0.89.

##### 2.2.2.3. The Impact of Event Scale (IES)

The 15-item IES [33, 34] measures the distressing impact of a particular event. The IES was administered to those students who indicated that they failed one or more exams and/or that they were disappointed by their results. Questions address the distressing impact, specifically of their latest exam results, in the past 7 days. Items are rated on a four-point scale, ranging from 0 (“not at all”), over 1 (“seldomly”), and 2 (“sometimes”), to 3 (“often”), and result in one sum score (although it is also possible to derive two subscale scores: “avoidance” and “intrusion”). The IES showed a very good reliability in the present sample: *α* = 0.93.

##### 2.2.2.4. The Generalization Scale of the Attitudes Toward Self Questionnaire (ATS-G)

The ATS-G [35] measures the tendency to generalize from a particular failure to the participant's sense of self-worth in four questions. For the present study, the ATS-G was adjusted to refer to their recently failed exams. Questions are rated on a 5-point scale, ranging from 1 (“strongly disagree”) to 5 (“strongly agree”). A sum score is calculated. Cronbach's alpha in the sample was acceptable: *α* = 0.70.

##### 2.2.2.5. Questionnaire NLEs (VNL)

The VNL [36] lists 31 distressing life events. These events are based on the NLE Questionnaire [37] and its more recent adaptation [38]. Two events were added to the original 31-item list used by Debeer, Hermans, and Raes [36]: “I ran into problems at my (vacation/volunteer) work,” “I had an unplanned pregnancy.” Participants indicated if these events occurred between Part 1 and Part 2 of the study (“yes”/“no”). Furthermore, items that received a positive answer are rated for their distressing impact on a VAS ranging from 0 (“did not suffer”) to 10 (“suffered very strongly”). This results in two sum scores: an unweighted sum score (0–33), reflecting the number of events, and a weighted sum score (0–330), reflecting the cumulative burden that is reported.

### 2.3. Procedure

#### 2.3.1. Part 1—Questionnaires and Conditioning Tasks

**Figure 2:**
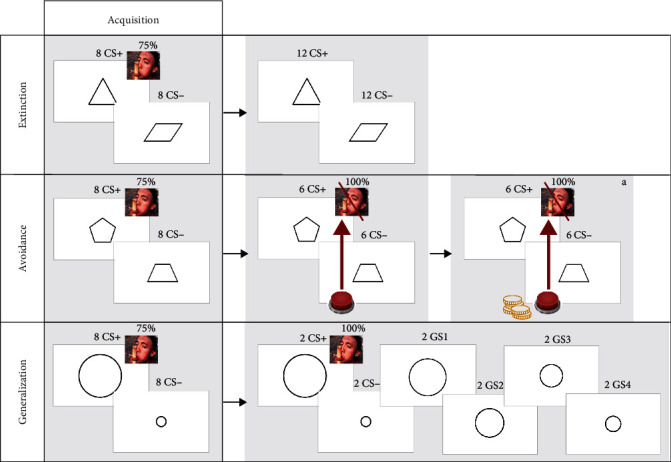
Procedure Part 1: conditioning tasks. ^a^Phase added after the first year of data collection.

The entire study took place online. Baseline assessments of anxiety and depression, as well as the conditioning tasks, were conducted on three consecutive days at the start of the academic year (in October). On the first day, after providing informed consent, participants filled out the DASS-21 and GAD-7. Subsequently, they underwent the conditioning tasks for that day (acquisition, followed by extinction, avoidance, or generalization). On the second and third day, participants completed the other conditioning tasks. The order of the conditioning tasks was counterbalanced across participants. An overview of the conditioning tasks can be found in Figure 2.

The trial flow was similar for all tasks: the experimental trials commenced with a 12-s presentation of the CS, accompanied by an expectancy rating scale (7 s) and subsequent distress rating scale (5 s). In trials where a US was present, it was presented for 1 s, immediately after the termination of the CS+. The intertrial interval lasted for 2 s. The order of the stimuli (CS and US) throughout all conditioning tasks was randomized before the start of the study but fixed across participants, with the aim of minimizing individual differences in the learning processes caused by differences in trial order.

Each conditioning task commenced with a *fear acquisition phase*. Before this phase started, participants received instructions that during the tasks of that day, geometric shapes would be shown that could sometimes be followed by an unpleasant image. They were further instructed that they would be asked to indicate to what extent they expected an image to follow. During the acquisition phase, the CS+ and CS− were each presented eight times. The CS+ was paired with a US in six trials, resulting in a 75% reinforcement rate. The CS− was never paired with a US.

The *extinction phase* commenced immediately after fear acquisition. No break or additional instructions were offered. The phase consisted of 24 trials in total: 12 CS+ trials and 12 CS− trials. Neither stimulus was paired with a US.

Before the *avoidance phase* started, participants were instructed that from that moment on, they would be able to prevent the presentation of the unpleasant image by pressing the spacebar in the first 2 s of the CS presentation. During this window, the words “spacebar in effect” appeared on the screen. Rating scales were presented immediately after. The avoidance phase consisted of six CS+ and six CS− trials. The CS+ was always paired with a US unless an avoidance response was performed on time. No cost was associated with the avoidance response. After the first year of data collection, an additional avoidance phase, including a cost, was added after the noncostly avoidance phase. This phase mimicked the original avoidance phase (six reinforced CS+ and six CS− trials) but had an additional instruction at the start: “From now on, each press of the spacebar will cost you one point. This point will be deducted from a total of 100 points.” Notably, the points were not associated with any other reward or outcome.

The *generalization phase* followed fear acquisition without any additional instructions or breaks. During generalization, four circles were introduced (GS1–GS4). These were intermediately sized between the CS+ and CS−, which were a small and a large circle [23]. The generalization phase consisted of 12 trials in total: two for each CS and GS. The CS+ was paired with a US in both of its trials.

#### 2.3.2. Part 2—Questionnaires

The second part of the study took place in February, 1 week after the participants were notified of their exam results for the first term. Emotional distress levels (anxiety, stress, and depression) were assessed by completing the DASS-21 and GAD-7. Subsequently, participants were asked if they failed any exams and if they were disappointed by these results. Those participants who indicated that they failed one or more exams and were disappointed with their exam results were asked to complete the ATS-G and IES. From the second year of data collection onwards, all participants additionally received the VNL to complete.

#### 2.3.3. Additional Follow-Up—IESs

Participants with disappointing failed exam results were asked to partake in an additional part, which commenced exactly 1 week after Part 2. This part consisted of the administration of the IES on a weekly basis for a duration of 10 weeks, allowing to assess individual differences in coping trajectories in response to the experience of failure.

#### 2.3.4. Part 3—Questionnaires

During Part 3 of the study (March), which took place exactly 1 month after Part 2, all participants were administered follow-up measures of the DASS-21 and GAD-7. Furthermore, the subset of participants with disappointing failed exams were asked to complete the IES and ATS-G.

### 2.4. Data Reduction

#### 2.4.1. Exclusions

Participants were excluded from the analyses on extinction, avoidance, or generalization, respectively, if they failed to show differential responses during fear acquisition on the corresponding day. Differential responding was determined as a higher average US-expectancy rating on the final three CS+ trials compared to the final three CS− trials of acquisition.

#### 2.4.2. Learning Indices

In order to relate responding during the conditioning tasks to questionnaire scores, seven learning indices were computed. These indices were designed to reflect different learning processes.

The *CS+ acquisition index* (ACQ_CS+_) reflected the average expectancy response toward the CS+ during the acquisition phase on the first day of the experiment. The *CS− acquisition index* (ACQ_CS+_) comprised the average expectancy response toward the CS− during that phase. The *early extinction index* (EXT_Early_) was determined by first calculating a “midway point” on a group level—specifically, the timepoint at which the expectancy ratings for the CS+ equaled the average of those observed on Trial 1 and Trial 12. In simpler terms, it represents the point, at the group level, where conditioned responding has halved. Subsequently, for each participant, the early extinction index was defined as the average expectancy rating to the CS+ during the trials immediately preceding and following that midway point, subtracted from their response on the first extinction trial. The index was then reverse-scored, so that higher values reflected reduced extinction from the start of the phase to the overall midway point. The *full extinction index* (EXT_Full_) was computed by averaging the expectancy ratings for the CS+ across the last two extinction trials and subtracting that value from the response on the first extinction trial, and then reverse scored as well, so that higher values reflected reduced whole-phase extinction. The *CS+ avoidance index* (AV_cs+_) was calculated by summing the number of CS+ trials during which the US was successfully avoided (i.e., spacebar pressed in the first 2 s of the trial) in the initial, no-cost avoidance phase. The *CS− avoidance index* (AV_cs−_) comprised the number of CS− trials (no-cost phase) where the spacebar was pressed in the first 2 s. Finally, we calculated a *generalization index* (GEN), which reflected overgeneralization to the GS. This index was calculated in a manner similar to Lenaert et al. [39]. The expectancy ratings from the initial trial of each GS were summed and then divided by the maximum response recorded across the initial trials of all GSs and CSs (i.e., range correction) (this GEN deviates from the original preregistered index. In that original index, GS scores were corrected for both CS+ and CS− responses $\left(\text{GEN}=\frac{\sum_{i=1}^{4}\left({\text{GS}}_{i}\right)-{\text{CS }}_{-}}{{\text{CS}}_{+}-{\text{CS }}_{-}}\right)$. However, the original index resulted in 64 extreme outliers [Q3 + 3 x IQR] for expectancy ratings [67 for distress ratings] due to unexpectedly low responses to the CS+ and/or unexpectedly high responses to the CS−. Therefore, it was replaced by the GEN proposed by Lenaert et al. [39]).

Six secondary indices were derived to conduct exploratory analyses. First, the index calculations for the acquisition, extinction, and generalization phases were repeated for the distress ratings, resulting in ACQ_Distress-CS+_, ACQ_Distress-CS−_, EXT_Distress-Early_, EXT_Distress-Full_, and GEN_Distress_. The midway point for EXT_Distress-Early_ was based on the expectancy ratings. Second, AV_Cost-CS+_ assessed the number of avoided CS+ trials in the costly avoidance phase. No AV_Cost-CS−_ was derived, as very few participants performed an avoidance response on any of the CS− trials (*N* = 32).

All indices were rescaled to *Z*-scores before they were included in further analyses.

### 2.5. Planned Statistical Analyses

#### 2.5.1. Manipulation Checks

First, we examined if fear conditioning across participants occurred as expected. For the *fear acquisition* phase, we employed a repeated measures analysis of variance (ANOVA), with Stimulus (CS+ and CS−) and Time (first trial and last trial) as within-subject variables. We expected a significant Stimulus x Time interaction effect. The fear *extinction* phase was similarly analyzed, anticipating a significant interaction effect between Time and Stimulus. We conducted a one-sample *t*-test on the no-cost *avoidance* phase to test if the number of avoided CS+ trials was significantly higher than zero. Additionally, we compared avoidance toward the CS+ versus the CS− using a paired-samples *t*-test. To assess overall *generalization*, we performed a repeated measures ANOVA on the first trial of each stimulus with Stimulus (CS+, CS−, GS1, GS2, GS3, GS4) as a within-subject variable, expecting a main effect for this variable.

#### 2.5.2. Interplay Learning Indices

Two sets of analyses were conducted to exploratorily assess the interplay of learning processes, as measured by our indices. First, we computed pairwise correlations between the indices. Where applicable, we used Pearson's correlation coefficient. In cases where the normality assumption was violated, Spearman's rank correlation was used. Second, to explore whether distinct learning profiles could be identified, a *k*-means cluster analysis was planned to be conducted across the main indices the original preregistration included only five main indices (reflecting extinction, avoidance, and generalization). Therefore, the cluster analysis was performed twice: once with the preregistered five indices and once with seven indices (including ACQ_CS+_ and ACQ_CS−_). Using a gap statistic, we determined the optimal number of clusters. This method compares the change in the within-cluster sum of squares over *k* = *n* solutions with that expected under an appropriate reference null distribution [40]. It helped to assess whether the observed clustering pattern was significantly different from a pattern that could be expected to appear by chance. We set the maximum *k* value to 10 and performed bootstrapping with 100 iterations to obtain reliable estimates.

#### 2.5.3. Correlations Between Learning Indices and Baseline Emotional Distress

To explore potential relationships between the seven learning indices and baseline (Part 1) levels of anxiety (DASS-A, GAD), stress (DASS-S), and depression (secondary analysis; DASS-D), we conducted a series of correlation analyses. Specifically, we examined the correlations between each of the seven indices and baseline (i.e., Part 1) levels of anxiety (DASS-A, GAD), stress (DASS-S), and depression (DASS-D). Pearson's correlation coefficient was used in cases where the assumption of normality was met. In the other cases, Spearman's rank correlation was used.

#### 2.5.4. Predicting Emotional Distress Over Time

To assess the prospective validity of the learning indices, we first investigated if they were predictive for changes in emotional distress throughout the sample. With this aim, we conducted a series of hierarchical linear regression analyses, predicting anxiety, stress, and depression levels 4 months (Part 2) to 5 months (Part 3) after the initial assessment. The predictors included the respective learning indices. Baseline anxiety, stress, or depression scores (questionnaires Part 1) were added as covariates in the analysis.

#### 2.5.5. Predicting the Impact of Failing Exams

Two sets of analyses were conducted to assess the prospective validity of learning indices for emotional distress after a specific stressor, namely failed exam results. The goal of the first set was to examine the effect of failure on emotional distress and, more importantly, to explore how the interaction between learning indices and failure affected emotional distress. We employed hierarchical models, in which we first added membership to the subgroup of students with disappointing failed exams as a predictor of emotional distress (anxiety, stress, depression, and self-generalization during Part 2 or 3), along with Part 1 questionnaire data as a covariate. Subsequently, we systematically added in individual learning indices and their interactions with the failed subgroup.

A second set of analyses on this topic explored if learning processes are associated with the psychological impact of the stressor over time. Specifically, we explored the relationship between the primary learning indices and the trajectories of IES scores within a subgroup of students who had faced disappointing failed exam results and were followed for 10 weeks after receiving their results. The analytical process comprised several key steps. We first plotted the course of the IES scores over time per participant and estimated four alternative latent growth models (linear, quadratic, cubic, logarithmic) to the dataset to describe their pattern. From these, the best-fitting model was selected on the basis of the Bayesian information criterion (BIC) and Akaike information criterion (AIC). Second, this best-fitting model was used as the foundation for constructing growth mixture models (GMMs) using the “lcmm” package in R [41]. We estimated both fixed- and random-effects GMM models, varying the number of subgroups from 1 to 6. Third, the optimal model was chosen based on the best log-likelihood value across 100 random starts, BIC, and AIC (deviating from our original preregistration, we chose not to employ the bootstrapped likelihood ratio test, as recommended by some researchers [42]. This decision was based on considerations of its computational intensity [43] and its inability to be integrated into the lcmm package) [44], providing insights into the underlying number of subgroups (Deviating from our original preregistration, we chose not to employ the Bootstrapped Likelihood Ratio Test, as recommended by some researchers [45]. This decision was based on considerations of its computational intensity [43] and its inability to be integrated into the lcmm package). Fourth, the participants were allocated to the different subgroups based on their highest posterior class probabilities. Finally, this subgroup membership was related to learning indices using a separate ANOVA for each learning index. A nonparametric Kruskall–Wallis test was used in cases where the normality assumption was violated.

#### 2.5.6. Predicting the Impact of NLE

To explore interactions between learning processes and life stressors beyond academic failure, we conducted exploratory analyses on the number and cumulative impact of NLE experienced between Part 1 and Part 2. The analyses mimicked those on the impact of failed exams: in the first step, the NLE index (number or cumulative impact) was added to the model containing baseline questionnaire data as a covariate. Second, individual learning indices were added in one by one, as well as their interactions with the NLE index.

Preregistration of the hypotheses and analyses is available at https://aspredicted.org/snjf-snt3.pdf.

## 3. Results

**Figure 3:**
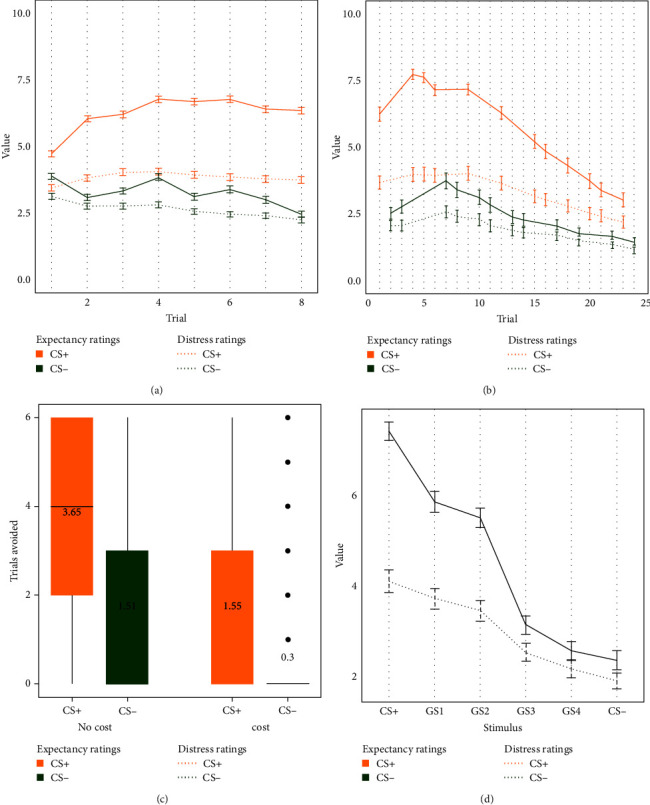
US-expectancy, distress, and avoidance responses throughout the phases: (A) acquisition over the 3 days; (B) extinction; (C) avoidance; (D) generalization. *Note*: Error bars represent the 95% confidence interval. CS, conditioned stimulus; US, unconditioned stimulus.

Graphs for US expectancies, distress ratings, and avoidance responses per stimulus and experimental phase are shown in Figure 3.

### 3.1. Manipulation Checks Conditioning Tasks

#### 3.1.1. Fear Acquisition

Across the 3 days, participants showed adequate fear learning, including clear discrimination between the CS+ and the CS−. This is supported by a significant Stimulus x Trial interaction, *F* (1, 1796) = 855.67, *p* < 0.001, *η*_p_^2^ = 0.32. Additionally, a significant main effect of Stimulus was found, *F* (1, 1796) = 1762.43, *p* < 0.001, *η*_p_^2^ = 0.50, but no significant main effect of Trial, *F* (1, 1796) = 2.62, *p*=0.106. The same analysis, conducted on distress ratings, revealed a significant interaction between Stimulus and Trial, *F* (1, 1736) = 271.48, *p* < 0.001, *η*_p_^2^ = 0.14, as well as significant effects of both factors: Stimulus, *F* (1, 1736) = 493.71, *p* < 0.001, *η*_p_^2^ = 0.22, and Trial, *F* (1, 1736) = 30.49, *p* < 0.001, *η*_p_^2^ = 0.02. Overall, these analyses suggest that (across participants and days) adequate acquisition learning took place. Analyses on a day-by-day and task-by-task level can be found in Supporting Information 1.

#### 3.1.2. Exclusion of Nonlearners

Based on the differential responding criterion (see Section 2.4.1), 14.89% of participants were excluded for the analyses on extinction, 25.24% for avoidance analyses, and 13.23% for generalization analyses.

#### 3.1.3. Extinction

As expected, during extinction, a significant Stimulus x Trial interaction emerged, *F* (1, 507) = 105.21, *p* < 0.001, *η*_p_^2^ = 0.17. Additionally, significant main effects of Stimulus, *F* (1, 507) = 414.16, *p* < 0.001, *η*_p_^2^ = 0.45, and Trial, *F* (1, 507) = 356.03, *p* < 0.001, *η*_p_^2^ = 0.41, were found. The same analyses, conducted on distress ratings, revealed a significant interaction between Stimulus and Trial, *F* (1, 487) = 24.32, *p* < 0.001, *η*_p_^2^ = 0.05, as well as significant main effects of Stimulus, *F* (1, 487) = 222.22, *p* < 0.001, *η*_p_^2^ = 0.31, and Trial, *F* (1, 487) = 168.38, *p* < 0.001, *η*_p_^2^ = 0.26. These results suggest that, overall, adequate extinction took place.

#### 3.1.4. Avoidance

On average, participants performed avoidance responses when confronted with the CS+ during the phase without a cost, *t* (464) = 36.14, *p* < 0.001, *d* = 1.68, as well as the phase with added cost, *t* (316) = 12.39, *p* < 0.001, *d* = 0.70. Exploratory analyses revealed that avoidance behavior in the presence of the CS+ was significantly higher compared to the CS−, both during the phase without cost, *t* (464) = 17.85, *p* < 0.001, *d* = 0.83, and during the phase with added cost, *t* (316) = 10.30, *p* < 0.001, *d* = 0.58.

#### 3.1.5. Generalization

Overall, participants demonstrated clear generalization of CRs. This was evidenced by a significant effect of Stimulus, *F* (5, 2605) = 425.67, *p* < 0.001, *η*^2^ = 0.37, in expectancy ratings provided during the first trial of each CS and GS. The same result was obtained for distress ratings: *F* (5, 2375) = 161.45, *p* < 0.001, *η*^2^ = 0.11.

### 3.2. Interplay Learning Indices

#### 3.2.1. Correlations Between Learning Indices

**Figure 4:**
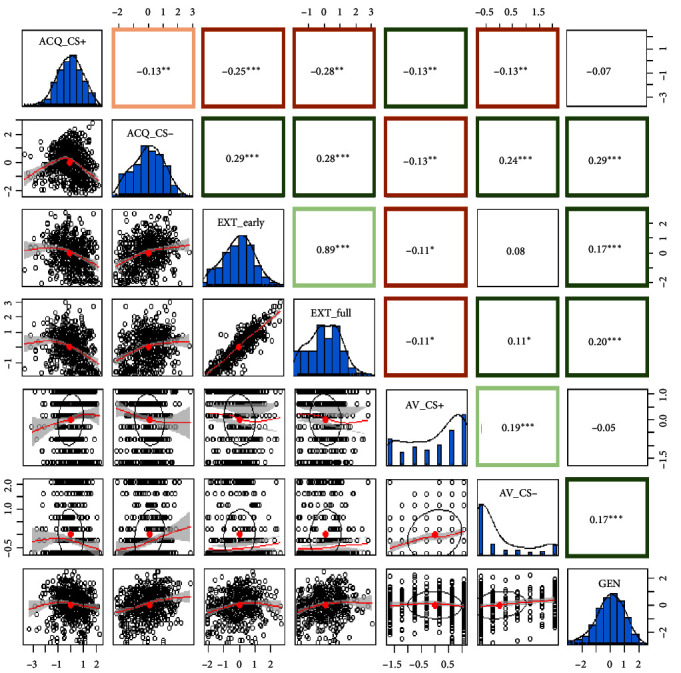
Correlations between primary indices (expectancy ratings and avoidance). ACQ_cs−, acquisition index CS− trials; ACQ_cs+, acquisition index CS+ trials; AV_ cs+, proportion avoided CS+ trials without cost; AV_cs−, proportion avoided CS− trials without cost; EXT_exp_early, early extinction index; EXT_exp_full, full extinction index; GEN, generalization index. *⁣*^*∗*^*p* < 0.05; *⁣*^*∗∗*^*p* < 0.01; *⁣*^*∗∗∗*^*p* < 0.001.

Pair plots (i.e., scatter plots, index distributions, as well as correlation parameters and their significance) assessing correlations between primary learning indices can be found in Figure 4.

As expected, due to their theoretical overlap and simultaneous measurement, primary indices based on the same phases were positively correlated for acquisition (negative correlation between CS+ and CS−; *p*=0.001), extinction (early and full extinction; *p* < 0.001), and avoidance (CS+ and CS− avoidance; *p* < 0.001).

Examining correlations between phases, the acquisition indices correlated significantly with all other indices. CS+ acquisition correlated with increased extinction (early and full, both *p* < 0.001), increased CS+ avoidance (*p*=0.005), and reduced CS− avoidance (*p*=0.005). CS− acquisition showed a reversed pattern, correlating significantly with reduced extinction (early and full, both *p* < 0.001), reduced (noncostly) CS+ avoidance (*p*=0.006), increased CS− avoidance (*p* < 0.001), and increased generalization (*p* < 0.001). Furthermore, increased generalization correlated significantly with reduced early and full extinction (both *p* < 0.001) and increased CS− avoidance (*p* < 0.001). Additionally, a smaller positive correlation was found between CS− avoidance and whole-phase extinction (*p*=0.029); increased avoidance was related to reduced whole-phase extinction. Finally, a small but significant negative correlation was found between CS+ avoidance and early (*p*=0.025) and whole-phase extinction (*p*=0.023); those who avoided the CS+ more, showed stronger extinction of expectancy ratings.

These same analyses were repeated for the secondary indices (those based on distress ratings; Supporting Information 2). Again, correlations between indices within a phase were found to be significant: for acquisition (a positive correlation in this case; *r* = 0.73, *p* < 0.001) and extinction (*r* = 0.83, *p* < 0.001). Assessing correlations between processes, CS+ related distress during acquisition correlated significantly with increased distress extinction (early, *r* = −0.17, *p* < 0.001; full, *r* = −0.24, *p* < 0.001), increased (noncostly) CS+ avoidance (*r* = 0.19, *p* < 0.001), and increased distress generalization (*r* = 0.16, *p* < 0.001). Similarly, CS− related distress during acquisition correlated significantly with increased whole-phase distress extinction (*r* = −0.09, *p*=0.033), increased avoidance (CS+, *r* = 0.10, *p*=0.039; CS−, *r* = 0.25, *p* < 0.001), and increased generalization (*r* = 0.34, *p* < 0.001). For the other processes, no significant correlations were found between distress extinction and avoidance or between distress extinction and distress generalization (all *p* > 0.05). Only the correlation between distress generalization and CS− avoidance (*r* = 0.14, *p*=0.004) was found to be significant.

In summary, the analyses revealed notable associations between acquisition, extinction, generalization, and avoidance. Different patterns were found for expectancy ratings (i.e., primary indices) and distress ratings (i.e., secondary indices). For indices based on expectancy ratings, reduced CS+ acquisition, extinction, and CS+ avoidance co-occurred with increased CS− acquisition, CS− avoidance, and generalization. For distress ratings, increased CS+ and CS− acquisition correlated with increased extinction, increased avoidance, and increased generalization. However, caution is warranted when interpreting correlations between CS+ acquisition and indices from the other processes. As acquiring a fear response to the CS+ was the starting point for all subsequent processes, their indices are inherently intertwined.

#### 3.2.2. Learning Profiles

The gap statistic identified a single cluster solution (*K* = 1) as the most suitable representation of the data (see Supporting Information 2) for indices based on expectancy ratings (i.e., primary indices) as well as distress ratings (i.e., secondary indices). This indicated that no distinct profiles could be identified in our sample based on the learning indices that were used.

### 3.3. Correlations Between Learning Indices and Baseline Emotional Distress

**Figure 5:**
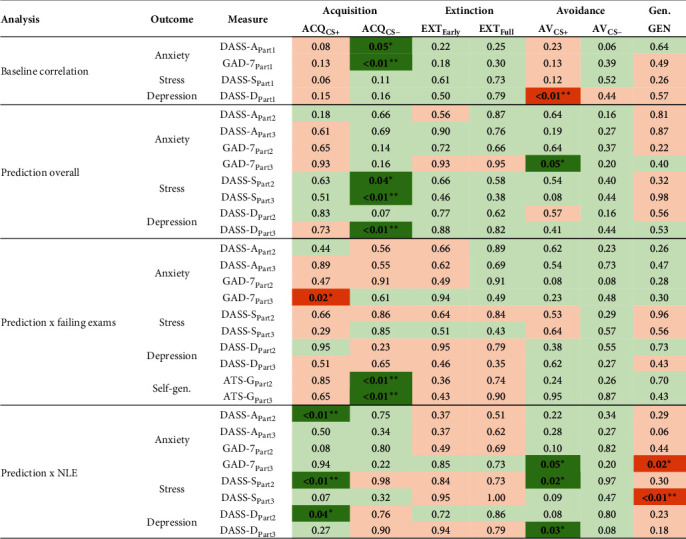
Correlations and hierarchical regression analyses for primary indices: resulting *p*-values. *Note:* Part 1 = baseline measurement of the corresponding questionnaire in October; Part 2 = postmeasurement in February (the corresponding analyses control for baseline responding); Part 3 = postmeasurement in March (the corresponding analyses control for baseline responding). Green fields = positive association (larger index − less favorable outcome). Orange fields = negative association (larger index − more favorable outcome). Bold values: significant (*p*  < 0.05, not corrected for multiple testing). ACQ_cs−_, acquisition index CS− trials based on expectancy ratings; ACQ_cs+_, acquisition index CS+ trials based on expectancy ratings; AV_cs−_, proportion avoided CS− trials without cost; AV_cs+_, proportion avoided CS+ trials without cost; DASS-A, DASS-S, DASS-D, subscales of the depression anxiety stress scales; EXT_Early_, early extinction index based on expectancy ratings; EXT_Full_, full extinction index based on expectancy ratings; GAD, generalized anxiety disorder seven-item scale; GEN, generalization index based on expectancy ratings. *⁣*^*∗*^*p* < 0.05; *⁣*^*∗∗*^*p* < 0.01; *⁣*^*∗∗∗*^*p* < 0.001.

Results for the correlation analyses between primary indices (i.e., expectancy ratings and noncostly avoidance) and baseline emotional distress measures are summarized at the top of Figure 5. Summarized results for the secondary indices (i.e., distress ratings and costly CS+ avoidance) are provided in Supporting Information 3. Comprehensive tables for both primary and secondary indices are provided in Supporting Information 4.

Notable correlations emerged between both primary and secondary indices of CS− acquisition and baseline anxiety (all *r* > 0.08, all *p* < 0.05); more anxious participants reported higher expectancies and stronger distress in response to the CS−. Furthermore, there was a significant negative correlation between noncostly CS+ avoidance and depression (*r* = −0.12, *p*=0.008): participants with higher levels of depression showed less CS+ avoidance. It is important to note that these values were not corrected for multiple testing due to the exploratory nature of the study.

The baseline questionnaires did not significantly correlate with primary or secondary indices of CS+ acquisition, early or full extinction, generalization, noncostly CS− avoidance, or costly CS+ avoidance (all *p* > 0.063).

### 3.4. Predicting Anxiety, Stress, and Depression Over Time

Overall, primary indices of CS+ acquisition, extinction, avoidance, nor generalization reliably predicted emotional distress after 4 (Part 2) to 5 (Part 3) months, controlling for baseline levels of the symptoms (see Figure 5 for an overview and Supporting Information 5 for detailed results). For these measures, only one small significant effect emerged: noncostly CS+ avoidance predicted anxiety in Part 3 of the experiment (but only as measured by the GAD-7; *p*=0.046). Conversely, CS− acquisition did predict changes in stress (Part 2, *p*=0.040; Part 3, *p*=0.004) and depression (Part 3, *p*=0.004), but not in anxiety.

For the secondary indices (distress ratings and costly avoidance; Supporting Information 3, 5), only costly CS+ avoidance emerged as a significant predictor of anxiety in Part 2 (but only as measured by the DASS-A; *p*=0.047).

Additionally, we assessed two-way interactions between the primary indices in the prediction of emotional distress (Supporting Information 5). Most significant interactions included one or both acquisition indices. Notably,*CS+ acquisition and CS− acquisition* reinforced each other in the prediction of anxiety (DASS-A, *p*=0.040) and stress (*p*=0.007) during Part 2. Increased *CS+ acquisition and decreased extinction* counterbalanced each other in the prediction of stress during Part 2 (early extinction, *p*=0.033; full extinction, *p*=0.084) and Part 3 (early extinction, *p*=0.019), and in the prediction of anxiety during Part 2 (GAD: early extinction, *p*=0.019; full extinction, *p*=0.006) during Part 2. Furthermore, a few mixed, less robust interaction effects emerged between *acquisition and avoidance*. CS+ acquisition and CS− avoidance reinforced each other in the prediction of anxiety (GAD, *p*=0.004) during Part 2. Conversely, CS+ acquisition and CS+ avoidance counterbalanced each other in the prediction of anxiety (DASS-A) in Part 2 (*p*=0.023) and Part 3 (*p*=0.038), and CS− acquisition and CS+ avoidance counterbalanced each other in the prediction of stress during Part 2 (*p*=0.045). It is important to note that most of these interactions only predicted anxiety and stress during Part 2. Fewer significant interactions emerged in the prediction of outcomes in Part 3, and no significant interactions emerged in the prediction of depression.

Out of 80 two-way interactions between the *other processes* (extinction, avoidance, and generalization), only two emerged as significant: noncostly CS+ avoidance and generalization reinforced each other in the prediction of stress (*p*=0.032) and depression (*p*=0.035) during Part 3.

### 3.5. Predicting the Impact of Failing Exams

#### 3.5.1. Anxiety, Stress, Depression, and Self-Generalization

The disappointing experience of receiving failed exam results emerged as a significant predictor for all questionnaire outcomes (controlled for baseline symptoms; Supporting Information 6), except for depression 1 month after receiving the results (Part 3; *p*=0.057). Overall, this suggests that encountering disappointing exams significantly negatively impacted emotional well-being, including anxiety, stress, depression, and generalization of the failure to sense of self-worth, both in the short term (1 week; Part 2) and in the long term (1 month; Part 3).

In a subsequent step, we introduced the individual learning indices to the model, as well as their interactions with the perceived failure (see Figure 5 for an overview and Supporting Information 6 for detailed results). From the seven primary indices, CS− acquisition emerged as a significant predictor of self-generalization after perceived failure (Part 2, *p*=0.002; Part 3, *p*=0.007), but not for anxiety, stress, or depression. Furthermore, in interaction with failure, stronger CS+ acquisition significantly predicted lower anxiety, but only as measured by the GAD-7 during Part 2 (*p*=0.02), and no significant effects were found on stress, depression, or self-generalization. For the primary extinction, avoidance, and generalization indices, no significant interactions were found with the experience of failure.

Similarly to the primary indices, from the six secondary indices (see Supporting Information 3, 6), CS− distress acquisition emerged as a significant predictor for stronger self-generalization after perceived failure (Part 2, *p*=0.022; Part 3, *p*=0.046), but not for any of the other outcome measures. Less robustly, in interaction with failure, reduced whole-phase distress extinction significantly predicted an increase in anxiety by Part 2 (*p*=0.035), but only as measured by the GAD-7 (not the DASS-A). Finally, the interaction between perceived failure and costly CS+ avoidance significantly predicted an increase in depression by Part 3 (*p*=0.016).

#### 3.5.2. IES Trajectories

A second set of analyses on this topic explored the association between the primary learning indices and the trajectories of IES scores within a sample of students with disappointing failed exam results (*N* = 216).

First, we identified a single-group growth model that best defined the dataset. We evaluated four potential models: linear, cubic, logarithmic, and quadratic. It became evident that the quadratic model outperformed the others in terms of model fit, as indicated by lower values of both the BIC and the AIC (Supporting Information 7). Additionally, the quadratic term (*x*^2^) was found to be highly significant (*p* < 0.001) within this quadratic model, distinguishing it from the cubic model, in which the cubic term (*x*³) did not attain statistical significance (*p*=0.316).

**Figure 6:**
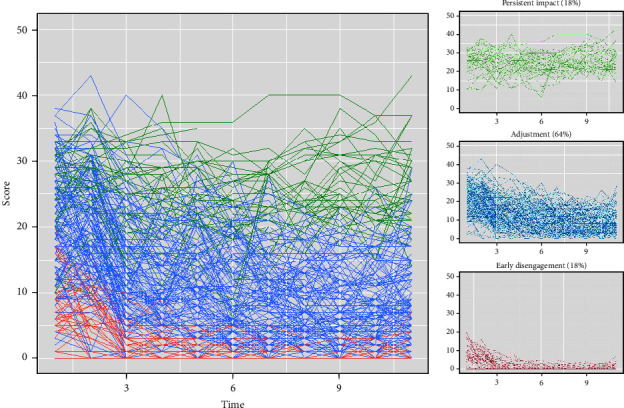
Participant impact of events trajectories with subgroup allocation. *Note*: Subgroup allocation based on a random intercepts and slopes GMM with three latent classes. GMM, growth mixture model.

Second, the quadratic model was used in a set of GMM models. Both fixed-effect (i.e., latent class growth analyses; LCGA) and random-effect (i.e., random intercepts, as well as random intercepts + slopes) models were fitted to the data, all with varying numbers of subgroups. From the 18 resulting models, the random intercepts and slopes model with three latent classes (i.e., subgroups) provided the lowest BIC and the most meaningful reduction in AIC and increase in log-likelihood and was therefore retained. Participants were allocated to the proposed three subgroups (Figure 6), which were then labeled “Persistent Impact,” “Adjustment,” and “Early Disengagement.”

Subsequent ANOVA's revealed that primary *acquisition* indices did not differ between IES trajectory groups; for CS+ expectancy acquisition, *F* (2, 211) = 0.98, *p*=0.337, nor CS− expectancy acquisition, *F* (2, 211) = 1.17, *p*=0.314. Secondary acquisition measures, however, did differ between trajectory groups. This was the case for both CS+ distress acquisition, *F* (2, 211) = 5.99, *p*=0.003, and CS− distress acquisition, *F* (2, 211) = 6.69, *p*=0.002. Post-hoc pairwise comparisons revealed that the Early Disengagement group reported lower distress acquisition to both CSs, compared to the Persistent Impact group (CS+, *p*_adj_ = 0.003; CS−, *p*_adj_ = 0.001) and compared to the Adjustment group (CS+, *p*_adj_ = 0.016; CS−, *p*_adj_ = 0.010). The Persistent Impact and Adjustment group did not differ significantly in CS responding (both *p* > 0.30). In summary, both CS+ and CS− distress acquisition were lowest in the group experiencing an early disengagement from the impact of failing exams.

Moreover, primary *avoidance* indices (i.e., noncostly CS+ and CS− avoidance) significantly differed between the trajectory groups, as evidenced by the nonparametric Kruskall–Wallis tests. Both noncostly CS− avoidance, *H* (2) = 9.37, *p*=0.009, *η*^2^ = 0.03, and noncostly CS+ avoidance, *H* (2) = 6.67, *p*=0.035, *η*^2^ = 0.02, differed significantly between the trajectory groups. Post hoc pairwise comparisons revealed that the Persistent Impact group avoided the CS− significantly more often compared to both the Early Disengagement (*p*_adj_ = 0.014) and Adjustment group (*p*_adj_ = 0.020). CS− avoidance did not differ significantly between the Early Disengagement and the Adjustment group (*p*_adj_ = 1.000). Additionally, these comparisons revealed that the Persistent Impact group avoided the CS+ significantly more often compared to the Early Disengagement group (*p*_adj_ = 0.030). Other comparisons were not significant (*p*_adj_ = 1.000). In summary, both CS+ and CS− avoidance were most common in the group experiencing a persistent negative impact of failing exams.

The primary *extinction* indices did not significantly differ between trajectory groups; for early extinction, *F* (2, 162) = 1.03, *p*=0.360, nor whole-phase extinction, *F* (2, 162) = 0.13, *p*=0.880. Neither did the primary GEN, *F* (2, 173) = 1.48, *p*=0.231, or any of their secondary indices (all *p*-values > 0.232).

### 3.6. Predicting the Impact of NLE

The number of NLE encountered between Part 1 and Part 2 emerged as a significant predictor for all questionnaire outcomes (controlling for baseline symptoms; Supporting Information 8). This suggests that the number of encountered NLE significantly impacted emotional distress, both measured in Part 2 and Part 3. The same was true for their cumulative burden.

In a subsequent step, we again introduced the learning indices to the model, as well as their interactions with the number of NLE (see Figure 5 and Supporting Information 8). Here, four noteworthy interactions were observed. First, in interaction with the number of NLE, *CS+ acquisition* emerged as a significant predictor of anxiety (DASS-A; *p*=0.008), stress (*p*=0.003), and depression (*p*=043) during Part 2. Second, in interaction with the number of NLE, noncostly *CS+ avoidance* significantly predicted higher stress during Part 2 (*p*=0.020), as well as anxiety (GAD-7; *p*=0.046) and depression (*p*=0.026) during Part 3. Third, and surprisingly, in interaction with the number of life events, *overgeneralization* significantly predicted lower stress (*p*=0.005) and anxiety (GAD-7; *p*=0.016) by Part 3. Fourth, no significant predictions were found based on CS− acquisition (early nor full) extinction or CS− avoidance. From the five secondary indices, only CS+ distress acquisition emerged as a significant predictor of anxiety (DASS-A, *p*=0.029) and stress (*p*=0.025) during Part 3.

Very similar results were found for the cumulative impact of NLE (see Supporting Information 8). In interaction with this impact, CS+ acquisition significantly predicted higher anxiety (DASS-A, *p*=0.015), stress (*p*=0.001), and depression (*p*=0.031) during Part 2, while CS− acquisition significantly predicted anxiety (DASS-A, *p*=0.040) in Part 3. Furthermore, CS+ avoidance significantly predicted higher stress during Part 2 (*p*=0.030) and depression during Part 3 (*p*=0.022), while overgeneralization significantly predicted lower stress (*p*=0.005) and anxiety (DASS-A; *p*=0.038, GAD-7; *p*=0.012) during Part 3. From the five secondary indices, unexpectedly, early distress extinction emerged as a significant predictor of lower anxiety during Part 3 (DASS-A, *p*=0.011) and lower stress during Part 2 (*p*=0.016).

## 4. Discussion

In this study, we addressed the question of why some individuals develop prolonged mental health problems in response to stressful life events while others do not, employing the diathesis-stress model as our theoretical framework. We focused on four learning processes—acquisition, extinction, avoidance, and generalization—identified as potential contributors to anxiety through their dynamic interactions with stressful life events. Our examination involved assessing verbal and behavioral measures of these learning processes and their interplay, with a focus on their predictive capacity for changes in emotional distress in first-year university students throughout the first 6 months of the academic year. This is a transitional phase, characterized by encounters with a broad set of stressors. In Part 1 (October), baseline questionnaires assessing emotional well-being were administered, as well as conditioning tasks. In Part 2 (February), we examined the stressors encountered by participants since Part 1, including perceived failure on exams and NLE. Additionally, emotional well-being was reassessed during both Parts 2 and 3 (March). Furthermore, throughout February, March, and April, we monitored individuals who experienced failed exams weekly to assess changes in the impact of that failure.

We initiated our analyses with an exploratory assessment of the interplay between individual learning processes at baseline. Clear relationships emerged among acquisition, extinction, avoidance, and generalization, substantiating the proposed connections between these fundamental processes. Different patterns emerged for indices based on expectancy ratings and distress ratings. For indices based on expectancy ratings, *reduced* CS+ acquisition, extinction, and CS+ avoidance co-occurred with *increased* CS− acquisition, CS− avoidance, and generalization. The former set of processes may all encompass a form of adaptive responding to (formerly) dangerous stimuli, while the latter set may share a deficit in safety learning [45]. For indices based on distress ratings, both increased CS+ and CS− acquisition correlated with increased extinction, increased avoidance, and increased generalization, suggesting a different shared characteristic (e.g., strength of fearful responding). However, caution is warranted when interpreting these findings, particularly regarding correlations between CS+ acquisition and other processes. As acquiring a fear response to the CS+ is foundational for all subsequent processes, their indices are unavoidably intertwined.

Contrary to expectations, our analyses could not support the existence of distinct profiles based on the examined vulnerability factors within the sample. This finding suggests that, despite their covariation, acquisition, extinction, avoidance, and generalization may not necessarily reinforce each other within individuals, leading to the absence of clear-cut learning profiles.

Subsequent analyses addressed our primary objective: examining if the four processes could predict anxiety. This was assessed in four ways. First, we evaluated whether individual learning indices correlated with baseline measures of emotional distress. From this analysis, CS− acquisition emerged as a robust predictor of stronger baseline anxiety. Furthermore, noncostly CS+ avoidance was found to be related to lower baseline depression.

Second, we assessed if the individual learning indices or their interactions could reliably predict changes in emotional distress over time in our sample, assuming that the proposed diatheses (learning indices) would interact with stressors experienced throughout the academic year. The results from these analyses suggested potential associations between increased CS− expectancy acquisition and worsening symptoms of stress and depression, and between CS+ avoidance and worsening symptoms of anxiety. Additionally, a few significant interactions emerged between the processes in the prediction of anxiety and stress. Most significant interactions occurred with CS+ (expectancy) acquisition. Combined with CS− acquisition and CS− avoidance, CS+ acquisition became more predictive for the development of anxiety and stress by Part 2. Conversely, in combination with CS+ avoidance and impaired extinction, it became less predictive for the development of anxiety and stress symptoms. Additionally, a significant interaction emerged between (noncostly) CS+ avoidance and generalization in predicting stress and depression, indicating a worsening of symptoms when both increased avoidance and overgeneralization were present. However, these findings were constrained by several factors: they were not robust across all outcome variables and timepoints and were found to have modest effect sizes.

Third, building upon the diathesis-stress model, we focused our attention on the interaction of learning processes with a specific stressor: the experience of failing exams. While the experience of failing exams significantly impacted emotional distress, most of our learning indices were not found to interact with that failure. Only CS− acquisition was found to be predictive for the self-generalizing impact of failed exams. However, a detailed exploration into coping trajectories during the weeks following the failure did uncover more notable associations: participants exhibiting strong (CS+ and CS−) distress acquisition were less likely to quickly emotionally disengage from their failure, while participants exhibiting frequent avoidance during the conditioning tasks were more likely to experience a persistent impact of the failure.

Fourth, over the course of our data collection, we introduced an additional questionnaire to explore the interaction between learning processes and a broader spectrum of NLE beyond failing exams. Similar to the consequences of failed exams, NLE emerged as a significant predictor of emotional distress. Furthermore, our investigation into the interaction between NLE and learning indices uncovered several noteworthy associations. In conjunction with NLE, CS+ acquisition, and CS+ avoidance were associated with heightened levels of stress, anxiety, and depression. Interestingly, overgeneralization predicted lower stress and anxiety, albeit only in the long term.

Together, these findings partially align with our initial expectations concerning the role of *acquisition*. In the prediction of symptoms, both CS+ acquisition and CS− acquisition turned out to be predictive of increases in emotional distress, albeit with some differences. CS− acquisition was found to be a rather robust predictor, being associated with baseline anxiety, as well as with a more sustained emotional impact of failing exams, and with the development of stress and depression, both by itself and in interaction with stressors. In contrast, CS+ acquisition was only found to be related to a sustained emotional impact of failing exams and to be a significant predictor of emotional distress in interaction with a set of NLE. These results align with preceding cross-sectional and prospective research, which increasingly supports the involvement of inadequate safety learning (i.e., CS− acquisition) in anxiety disorders, whereas evidence for the role of CS+ acquisition remains mixed [7, 9, 10].

Moreover, these findings align with our initial expectations concerning *avoidance* behaviors as predictors of anxiety. In cross-sectional research, excessive avoidance has been shown to be consistently overrepresented in patients with anxiety disorders [19, 20]. Our findings extend this understanding by offering some of the first evidence that avoidance of a conditioned threat stimulus in a fear conditioning task may indeed serve as a predictive indicator for the onset of anxiety and by extension, emotional distress. However, again, a caveat remains concerning robustness: similarly to CS+ acquisition, CS+ avoidance was only somewhat robust as a predictor of emotional distress.

Regarding *extinction* and *generalization*, our initial expectations were not met. The evidence did not strongly support the hypothesis that impaired extinction at baseline predicts the emergence of emotional distress. This finding diverges from earlier studies conducted by Guthrie and Bryant [12], Lommen et al. [13, 15], and Orr et al. [14]. Similarly, our evidence failed to support the hypothesis that overgeneralization predicts the emergence of emotional distress over time, as observed by Lenaert et al. [5], despite employing a highly similar sample and design.

The lack of support for the hypotheses concerning extinction and generalization prompts a cautious interpretation. These nonsignificant findings may suggest that extinction and generalization measured in conditioning tasks cannot robustly predict the onset of emotional distress. The large sample size enhances confidence in our results, suggesting that our study was equipped to detect meaningful effects if they existed. However, interpreting nonsignificance also prompts a careful examination of the methodology that was used. In the following paragraphs, we highlight two factors that warrant attention in future research.

First, our departure from earlier research findings may stem from *procedural differences* in fear conditioning designs. Researchers currently employ diverse designs when studying fear conditioning, introducing significant variability in factors such as procedure duration, reinforcement rates, stimulus types, and outcome measures. Little is known about the impact of such procedural aspects on the indices and their reflection of the underlying processes they aim to assess. Therefore, to gain a more comprehensive understanding of the intricate relationship between conditioning indices and psychological outcomes, a nuanced exploration incorporating procedurally diverse designs may be crucial.

A concrete suggestion based on this limitation would be to replicate the current design in a laboratory setting, facilitating some procedural variation. For one, because our study was conducted online, we exclusively relied on verbal ratings and avoidance behavior to assess fear. While these are recognized as valid indicators of fear [46], they only address two of the three key response systems typically involved in fear: subjective apprehension and avoidance behavior [47]. The third system, physiological arousal, was not directly assessed in our study. Although some research suggests that physiological arousal can be effectively captured through self-report measures like distress ratings [46], replicating this study in a laboratory setting would provide the possibility to directly assess physiological measures of fear. Additionally, emotionally aversive IAPS pictures [27] were used as USs. Although these USs successfully elicited aversive learning, as indicated by the fear and avoidance responses throughout the tasks, future laboratory studies could use more physically salient USs (e.g., electric or auditory stimulation), potentially resulting in more robust predictions.

A second potential limitation stems from the *choice of learning indices*. The decision to use these indices was in part driven by an aim to ensure replicability in future research and to enhance consistency with existing literature. Two specific limitations relate to this decision. First, it is important to acknowledge that currently, only theoretical arguments and use in former research [5, 13, 15] guide us in determining which indices may reflect the underlying processes better, leading to some arbitrariness in their choice. Second, there is a risk that using aggregate indices may result in overlooking important details or nuances in the data, leading to the potential loss of valuable information. For instance, in the construction of extinction indices, we focused on specific trials rather than overall patterns. Adopting a more data-driven approach, such as LCGA or GMM to identify learning patterns, could potentially overcome both limitations. While this alternative approach may compromise some replicability, it offers a more detailed understanding, particularly in the contexts of extinction and generalization. Alternatively, conducting a multiverse analysis to assess the impact of choices made in the construction of indices on the significance of predictive results may provide more insight into which aspects of conditioning tasks are predictive of emotional distress.

## 5. Conclusions

In summary, our study offers valuable insights into the roles of acquisition, extinction, avoidance, and generalization in emotional distress following exposure to stressors. This study marks the first comprehensive examination of these four processes together. While we did find correlations among them, we did not observe clear evidence of mutual reinforcement within individuals, as indicated by the absence of distinct learning profiles. Predictive analyses revealed that the acquisition of fear regarding safe stimuli (CS−), as well as the acquisition of fear and avoidance regarding dangerous stimuli (CS+), may be predictive for the development of emotional distress. However, it is crucial to acknowledge the limited robustness, especially concerning CS+ acquisition and avoidance, as well as the overall lack of significant findings concerning extinction and overgeneralization. These findings underscore the need for future research to explore the impact of methodological choices on significance, as well as the need to consider more data-driven approaches, ensuring a more comprehensive understanding of the impact of anxiety-related processes on well-being.

## Data Availability

Preregistration of the hypotheses and analyses is available at https://aspredicted.org/snjf-snt3.pdf. The participant data and analysis scripts are available at https://osf.io/zjy6h/. Finally, the Questionnaire Negative Life Events [36] used in this study can be made available upon request.

## Supporting Information

Supporting Information Additional results of data analysis, figures, and tables mentioned in this manuscript are provided in the supporting information listed below. Supporting Information 1. Additional manipulation checks acquisition. Supporting Information 2. Interplay learning indices. Supporting Information 3. Overview table—secondary indices. Supporting Information 4. Correlations between learning indices and baseline emotional distress. Supporting Information 5. Predicting anxiety, stress, and depression (full tables). Supporting Information 6. Predicting the impact of failing exams (full tables). Supporting Information 7. Impact of failing exams: trajectories (growth mixture modeling). Supporting Information 8. Predicting the impact of negative life events (full tables).
