# Supplementary material for: Predicting Emotional Distress, Based on Acquisition, Extinction, Avoidance, and Generalization Learning
Source: Depress Anxiety. 2024 Oct 23;2024:6366269. doi: 10.1155/2024/6366269 (PMC11918936; doi:10.1155/2024/6366269)
Supplement: Supporting Information — Additional results of data analysis, figures, and tables mentioned in this manuscript are provided in the supporting information listed below. Supporting Information 1. Additional manipulation checks acquisition. Supporting Information 2. Interplay learning indices. Supporting Information 3. Overview table—secondary indices. Supporting Information 4. Correlations between learning indices and baseline emotional distress. Supporting Information 5. Predicting anxiety, stress, and depression (full tables). Supporting Information 6. Predicting the impact of failing exams (full tables). Supporting Information 7. Impact of failing exams: trajectories (growth mixture modeling). Supporting Information 8. Predicting the impact of negative life events (full tables). [file 6366269.f1.docx]

## Supplementary Material 1 – Additional Manipulation Checks Acquisition

**Table 1**

*Manipulation check acquisition on a day-by-day level*

| Measure | Day | Effect | *df_n_* | *df_d_* | *F* | *p* | η_p_^2^ |
| --- | --- | --- | --- | --- | --- | --- | --- |
| Expectancy | 1 | Stimulus | 1 | 622 | 385.54 | <.001*** | 0.38 |
|  |  | Trial | 1 | 622 | 11.38 | <.001*** | 0.02 |
|  |  | Stimulus x Trial | 1 | 622 | 212.01 | <.001*** | 0.25 |
|  | 2 | Stimulus | 1 | 605 | 763.29 | <.001*** | 0.56 |
|  |  | Trial | 1 | 605 | 0.00 | .994 | 0.00 |
|  |  | Stimulus x Trial | 1 | 605 | 331.60 | <.001*** | 0.35 |
|  | 3 | Stimulus | 1 | 567 | 705.20 | <.001*** | 0.55 |
|  |  | Trial | 1 | 567 | 0.68 | .410 | 0.00 |
|  |  | Stimulus x Trial | 1 | 567 | 333.79 | <.001*** | 0.37 |
| Distress | 1 | Stimulus | 1 | 599 | 109.69 | <.001*** | 0.16 |
|  |  | Trial | 1 | 599 | 5.65 | .018* | 0.01 |
|  |  | Stimulus x Trial | 1 | 599 | 60.52 | <.001*** | 0.09 |
|  | 2 | Stimulus | 1 | 582 | 197.89 | <.001*** | 0.25 |
|  |  | Trial | 1 | 582 | 9.70 | .002** | 0.02 |
|  |  | Stimulus x Trial | 1 | 582 | 113.00 | <.001*** | 0.16 |
|  | 3 | Stimulus | 1 | 553 | 196.86 | <.001*** | 0.26 |
|  |  | Trial | 1 | 553 | 20.04 | <.001*** | 0.04 |
|  |  | Stimulus x Trial | 1 | 553 | 111.53 | <.001*** | 0.17 |

**Table 2**

*Manipulation check acquisition on a phase-by-phase level*

| Measure | Subsequent phase | Effect | *df_n_* | *df_d_* | *F* | *p* | η_p_^2^ |
| --- | --- | --- | --- | --- | --- | --- | --- |
| Expectancy | Extinction | Stimulus | 1 | 589 | 722.52 | <.001*** | 0.55 |
|  |  | Trial | 1 | 589 | 13.29 | <.001*** | 0.02 |
|  |  | Stimulus x Trial | 1 | 589 | 395.99 | <.001*** | 0.40 |
|  | Avoidance | Stimulus | 1 | 603 | 426.91 | <.001*** | 0.42 |
|  |  | Trial | 1 | 603 | 26.98 | <.001*** | 0.04 |
|  |  | Stimulus x Trial | 1 | 603 | 229.79 | <.001*** | 0.28 |
|  | Generalization | Stimulus | 1 | 602 | 662.95 | <.001*** | 0.52 |
|  |  | Trial | 1 | 602 | 2.14 | .144 | 0.00 |
|  |  | Stimulus x Trial | 1 | 602 | 254.38 | <.001*** | 0.30 |
| Distress | Extinction | Stimulus | 1 | 576 | 168.31 | <.001*** | 0.23 |
|  |  | Trial | 1 | 576 | 35.40 | <.001*** | 0.06 |
|  |  | Stimulus x Trial | 1 | 576 | 79.26 | <.001*** | 0.12 |
|  | Avoidance | Stimulus | 1 | 581 | 119.11 | <.001*** | 0.17 |
|  |  | Trial | 1 | 581 | 0.65 | 0.419 | 0.00 |
|  |  | Stimulus x Trial | 1 | 581 | 123.88 | <.001*** | 0.18 |
|  | Generalization | Stimulus | 1 | 577 | 215.06 | <.001*** | 0.27 |
|  |  | Trial | 1 | 577 | 6.70 | 0.010* | 0.01 |
|  |  | Stimulus x Trial | 1 | 577 | 70.50 | <.001*** | 0.11 |

## Supplementary Material 2 – Interplay Learning Indices

**Figure 1**

*Correlations Between Secondary Indices (Distress Ratings) and Avoidance*

*
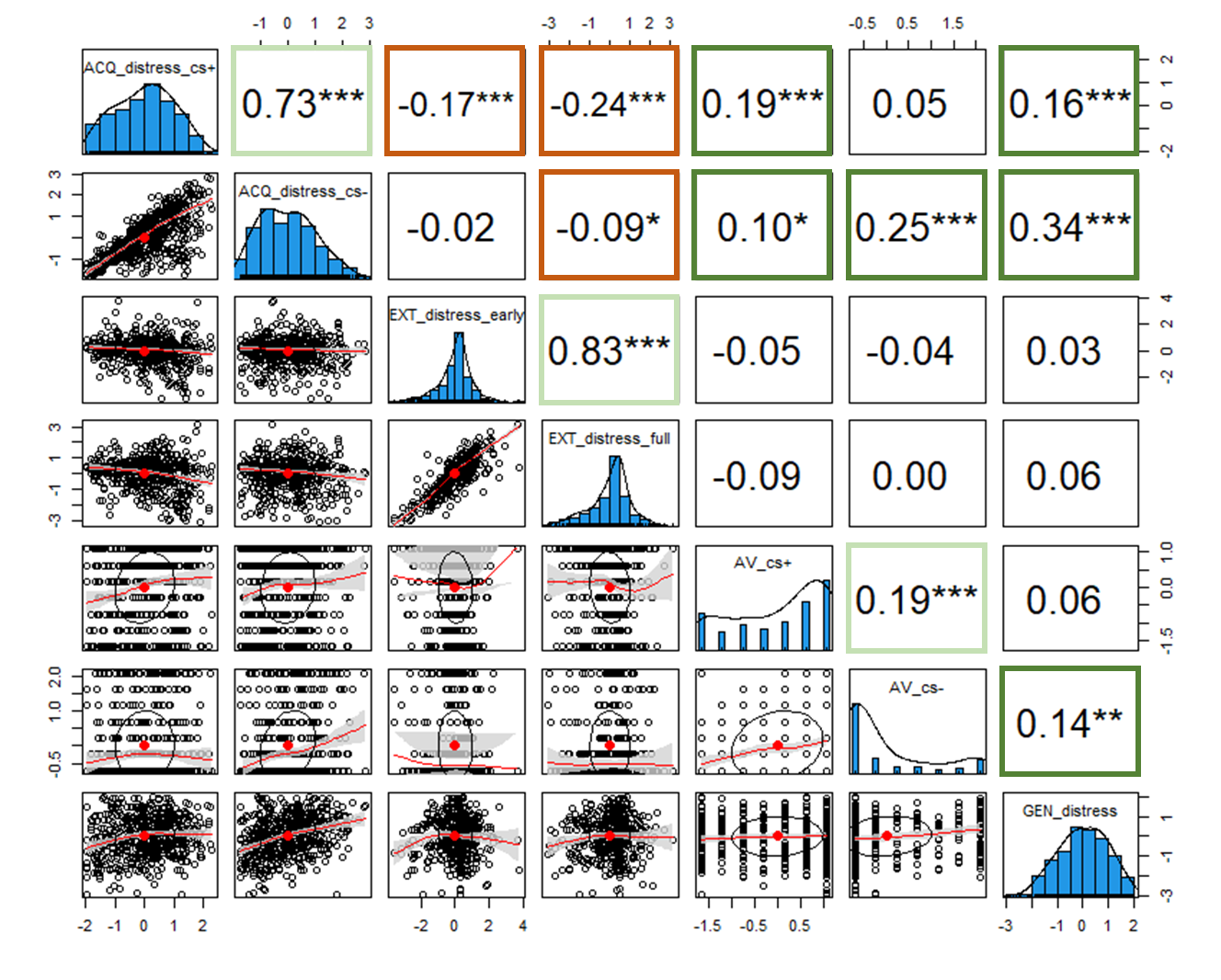
*

*Note*. ACQ_distress_CS+ = acquisition index CS+ trials based on distress ratings, ACQ_distress_CS- = acquisition index CS- trials based on distress ratings, EXT_distress_early = early extinction index distress ratings, EXT_ distress _full = full extinction index distress ratings, AV_CS+ = proportion avoided CS+ trials without cost, AV_CS- = proportion avoided CS- trials without cost, GEN_ distress = generalization index

**Figure 2**

*Gap Statistic for K-Means Cluster Analyses with an Increasing Number of Clusters (Primary Indices: Expectancy Ratings and Avoidance, Excluding Acquisition Indices)*


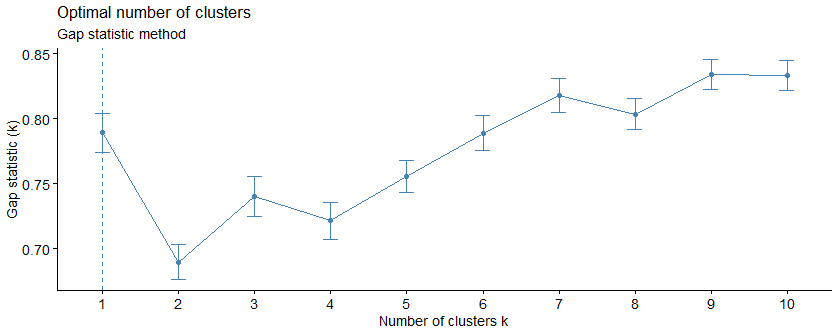


**Figure 3**

*Gap Statistic for K-Means Cluster Analyses with an Increasing Number of Clusters (Primary Indices: Expectancy Ratings and Avoidance, Including Acquisition Indices)*


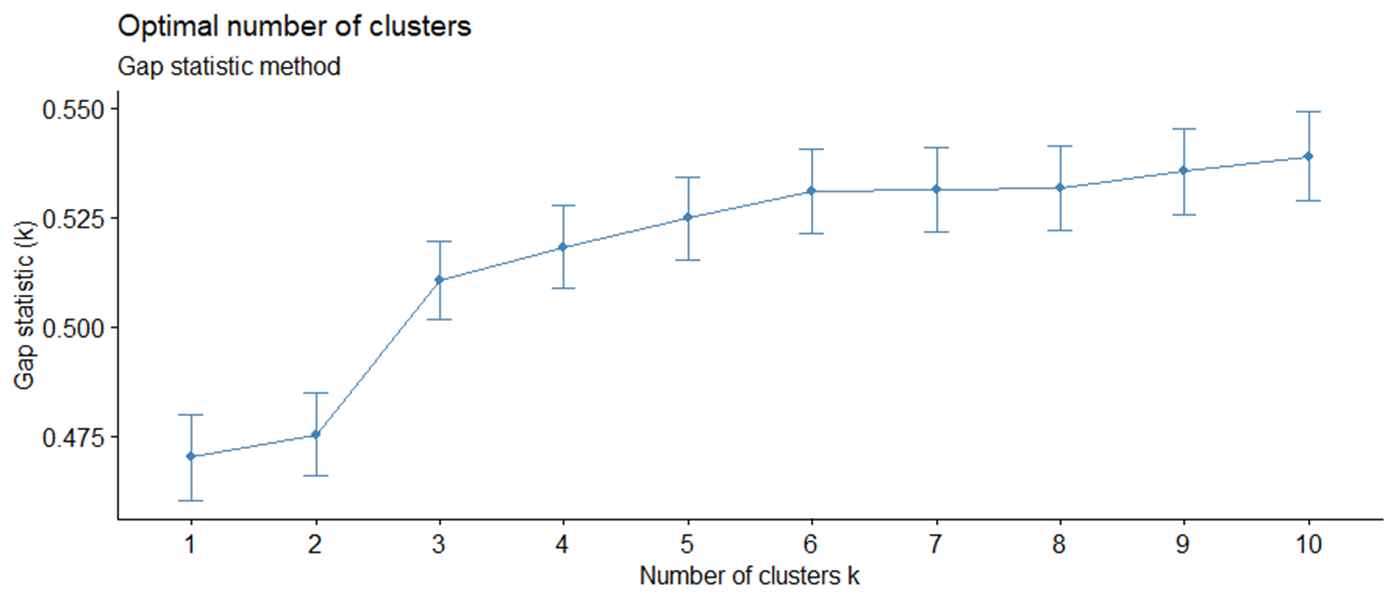


**Figure 4**

*Gap Statistic for K-Means Cluster Analyses with an Increasing Number of Clusters (Secondary Indices: Distress Ratings and Avoidance, Including Acquisition Indices)*


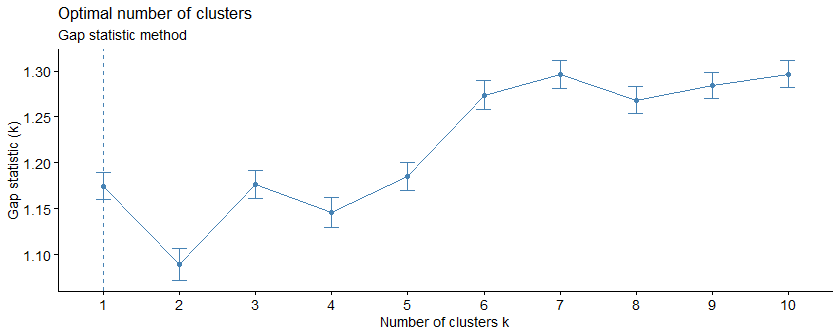


**Figure 5**

*Gap Statistic for K-Means Cluster Analyses with an Increasing Number of Clusters (Secondary Indices: Distress Ratings and Avoidance, Including Acquisition Indices)*


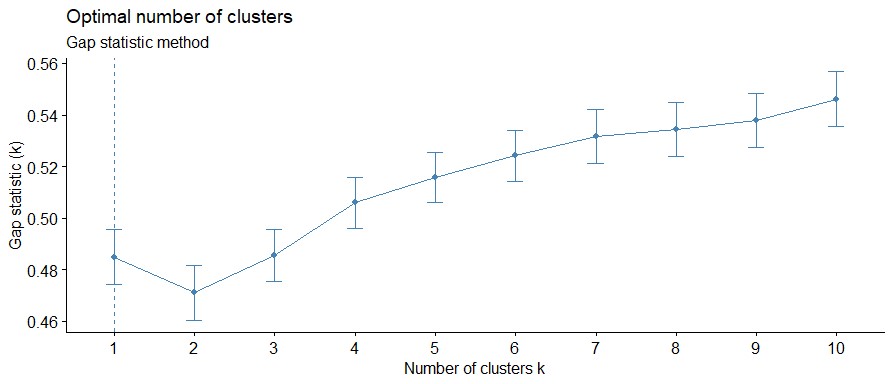


## Supplementary Material 3 – Overview Table – Secondary Indices

|  |  |  | Acquisition | | Extinction | | Avoidance | Gen. |
| --- | --- | --- | --- | --- | --- | --- | --- | --- |
|  |  |  | ACQ_Dist-CS+_ | ACQ_Dist-CS-_ | EXT_Dist-Early_ | EXT_Dist-Full_ | AV_Cost-CS+_ | GEN_Dist_ |
| Baseline corr. | Anxiety | DASS-A_Part1_ | .14 | **< .01**** | .86 | .40 | .71 | .80 |
|  |  | GAD-7_Part1_ | .09 | **< .01**** | .60 | .95 | .69 | .30 |
|  | Stress | DASS-S_Part1_ | .21 | .09 | .92 | .51 | .47 | .82 |
|  | Depression | DASS-D_Part1_ | .93 | .10 | .79 | .97 | .31 | .39 |
|  |  |  |  |  |  |  |  |  |
| Prediction overall | Anxiety | DASS-A_Part2_ | .15 | .58 | .99 | .17 | **.05*** | .52 |
|  |  | DASS-A_Part3_ | .47 | .52 | .68 | .83 | .77 | .68 |
|  |  | GAD-7_Part2_ | .77 | .62 | 1.00 | .33 | .93 | .72 |
|  |  | GAD-7_Part3_ | .73 | .78 | .92 | .42 | .57 | .64 |
|  | Stress | DASS-S_Part2_ | .23 | .12 | .78 | .67 | .76 | .77 |
|  |  | DASS-S_Part3_ | .26 | .31 | .54 | .96 | .48 | .64 |
|  | Depression | DASS-D_Part2_ | .67 | .34 | .66 | .66 | .10 | .92 |
|  |  | DASS-D_Part3_ | .68 | .31 | .76 | .98 | .24 | .75 |
|  |  |  |  |  |  |  |  |  |
| Prediction x failing exams | Anxiety | DASS-A_Part2_ | .06 | .55 | .99 | .55 | .06 | .50 |
|  |  | DASS-A_Part3_ | .23 | .57 | .29 | .41 | .68 | .23 |
|  |  | GAD-7_Part2_ | .85 | .44 | .56 | .83 | .48 | .21 |
|  |  | GAD-7_Part3_ | .43 | .74 | .08 | **.04*** | .70 | .16 |
|  | Stress | DASS-S_Part2_ | .64 | .51 | .55 | .82 | .43 | .19 |
|  |  | DASS-S_Part3_ | .47 | .99 | .47 | .17 | .38 | .09 |
|  | Depression | DASS-D_Part2_ | .91 | .48 | .18 | .83 | **.02*** | .54 |
|  |  | DASS-D_Part3_ | .52 | .60 | .94 | .90 | .55 | .43 |
|  | Self-gen. | ATS-G_Part2_ | .57 | **.02*** | .72 | .53 | .90 | .12 |
|  |  | ATS-G_Part3_ | .31 | **.05*** | .98 | .93 | .14 | .20 |
|  |  |  |  |  |  |  |  |  |
| Prediction x NLE | Anxiety | DASS-A_Part2_ | .09 | .33 | .18 | .34 | .30 | .46 |
|  |  | DASS-A_Part3_ | **.03*** | .07 | .29 | .79 | .28 | .37 |
|  |  | GAD-7_Part2_ | .41 | .63 | .63 | .88 | .80 | .73 |
|  |  | GAD-7_Part3_ | .83 | .91 | .72 | .39 | .68 | .75 |
|  | Stress | DASS-S_Part2_ | .19 | .56 | .07 | .12 | .22 | .24 |
|  |  | DASS-S_Part3_ | .28 | .68 | .61 | .61 | .30 | .70 |
|  | Depression | DASS-D_Part2_ | .39 | .74 | .90 | .81 | .10 | .39 |
|  |  | DASS-D_Part3_ | **.03*** | .07 | .34 | .41 | .22 | .33 |

*Note*. DASS-A, DASS-S, DASS-D = subscales of the Depression Anxiety Stress Scales; GAD = Generalized Anxiety Disorder seven item scale; ACQ_Dist-CS+_ = acquisition index CS+ trials based on distress ratings; ACQ_Dist-CS-_ = acquisition index CS- trials based on distress ratings; EXT_Dist-Early_ = early extinction index based on distress ratings; EXT_Dist-Full_ = full extinction index based on distress ratings; AV_Cost-CS+_ = proportion avoided CS+ trials with cost, GEN_Dist_ = generalization index based on distress ratings. Part 1 = baseline measurement of the corresponding questionnaire in October; Part 2 = post-measurement in February (the corresponding analyses control for baseline responding); Part 3 = post-measurement in March (the corresponding analyses control for baseline responding)

Green fields = positive association (larger index ~ less favorable outcome). Orange fields= negative association (larger index ~ more favorable outcome). Bold values: significant (p < .05, not corrected for multiple testing).

## Supplementary Material 4 – Correlations between Learning Indices and Baseline Emotional Distress

**Table 1**

*Correlations Between Baseline Emotional Distress Measures (Anxiety, Stress, and Depression) and Learning* *Indices*

| **Primary Indices** | | | |  | **Secondary Indices** | | | |
| --- | --- | --- | --- | --- | --- | --- | --- | --- |
|  | ***df*** | ***r*** | ***p*** |  |  | ***df*** | ***r*** | ***p*** |
| **DASS-A** |  |  |  |  |  |  |  |  |
| ACQ_cs+_ | 627 | -.07 | .083 |  | ACQ_Dist-csplus_ | 627 | .06 | .143 |
| ACQ_cs-_ | 627 | .08 | .050* |  | ACQ_Dist-csmin_ | 627 | .11 | .004** |
| EXT_Early_ | 503 | .05 | .225 |  | EXT_Dist-Early_ | 495 | .01 | .858 |
| EXT_Full_ | 503 | .05 | .254 |  | EXT_Dist-Full_ | 494 | .04 | .403 |
| AV_nocost-cs-_ | 451 | -.06 | .232 |  | AV_cost-csplus_ | 306 | -.02 | .712 |
| AV_nocost-cs+_ | 451 | .09 | .063 |  | GEN_Dist_ | 522 | .01 | .803 |
| GEN | 521 | .02 | .638 |  |  |  |  |  |
| **DASS-S** |  |  |  |  |  |  |  |  |
| ACQ_csplus_ | 627 | -.08 | .056 |  | ACQ_Dist-csplus_ | 627 | .05 | .207 |
| ACQ_csmin_ | 627 | .06 | .114 |  | ACQ_Dist-csmin_ | 627 | .07 | .085 |
| EXT_Early_ | 503 | .02 | .613 |  | EXT_Dist-Early_ | 495 | .00 | .923 |
| EXT_Full_ | 503 | .02 | .730 |  | EXT_Dist-Full_ | 494 | .03 | .508 |
| AV_nocost-csmin_ | 451 | -.07 | .116 |  | AV_cost-csplus_ | 306 | .04 | .468 |
| AV_nocost-csplus_ | 451 | .03 | .520 |  | GEN_Dist_ | 522 | -.01 | .820 |
| GEN | 521 | -.05 | .256 |  |  |  |  |  |
| **DASS-D** |  |  |  |  |  |  |  |  |
| ACQ_csplus_ | 627 | -.06 | .148 |  | ACQ_Dist-csplus_ | 627 | .00 | .935 |
| ACQ_csmin_ | 627 | .06 | .156 |  | ACQ_Dist-csmin_ | 627 | .06 | .103 |
| EXT_Early_ | 503 | .03 | .496 |  | EXT_Dist-Early_ | 495 | -.01 | .793 |
| EXT_Full_ | 503 | .01 | .785 |  | EXT_Dist-Full_ | 494 | .00 | .967 |
| AV_nocost-csmin_ | 451 | -.12 | .008** |  | AV_cost-csplus_ | 306 | -.06 | .312 |
| AV_nocost-cs+_ | 451 | -.04 | .439 |  | GEN_Dist_ | 522 | -.04 | .388 |
| GEN | 521 | -.02 | .574 |  |  |  |  |  |
| **GAD-7** |  |  |  |  |  |  |  |  |
| Acq_csplus_ | 627 | -.06 | .132 |  | ACQ_Dist-csplus_ | 627 | .07 | .088 |
| Acq_csmin_ | 627 | .12 | .003** |  | ACQ_Dist-csmin_ | 627 | .12 | .002** |
| EXT_Early_ | 503 | .06 | .182 |  | EXT_Dist-Early_ | 495 | -.02 | .596 |
| EXT_Full_ | 503 | .05 | .297 |  | EXT_Dist-Full_ | 494 | .00 | .950 |
| AV_nocost-csmin_ | 451 | -.07 | .127 |  | AV_cost-csplus_ | 306 | .02 | .687 |
| AV_nocost-csplus_ | 451 | .04 | .390 |  | GEN_Dist_ | 522 | -.05 | .303 |
| GEN | 521 | -.03 | .494 |  |  |  |  |  |

*Note*. DASS-A, DASS-S, DASS-D = subscales of the Depression Anxiety Stress Scales; GAD = Generalized Anxiety Disorder seven item scale; ACQ_csplus_ = acquisition index CS+ trials based on expectancy ratings; ACQ_csmin_ = acquisition index CS- trials based on expectancy ratings; EXT_Early_ = early extinction index based on expectancy ratings; EXT_Full_ = full extinction index based on expectancy ratings; AV_nocost_csmin_ = proportion avoided CS- trials without cost, AV_nocost_csplus_ = proportion avoided CS+ trials without cost, GEN = generalization index based on expectancy ratings. ACQ_Dist-csplus_ = acquisition index CS+ trials based on distress ratings; ACQ_Dist-csmin_ = acquisition index CS- trials based on distress ratings; EXT_Dist-Early_ = early extinction index based on distress ratings; EXT_Dist-Full_ = full extinction index based on distress ratings; AV_cost_csplus_ = proportion avoided CS+ trials without cost, GEN_Dist_ = generalization index based on distress ratings.

*** *p* < .001; ** *p* < .01; * *p* < .05

## Supplementary Material 5 – Predicting Anxiety, Stress, and Depression (Full Tables)

**Table 1**

*Prediction of Anxiety, Stress, And Depression from Primary Learning* *Indices*

|  | **Part 2** | | | | **Part 3** | | | |
| --- | --- | --- | --- | --- | --- | --- | --- | --- |
|  | **∆*R^2 a^*** | **β** | ***t*** | ***p*** | **∆*R^2 a^*** | **β** | ***t*** | ***p*** |
| **DASS-A** |  |  |  |  |  |  |  |  |
| Baseline | .36 | 0.61 | 17.55 | < .001*** | .34 | 0.60 | 16.60 | < .001*** |
| ACQ_cs+_ | .00 | 0.20 | 1.35 | .179 | .00 | -0.08 | -0.52 | .606 |
| ACQ_cs-_ | .00 | 0.07 | 0.44 | .657 | .00 | 0.06 | 0.40 | .691 |
| EXT_Early_ | .00 | -0.09 | 0.59 | .557 | .00 | 0.02 | -0.13 | .899 |
| EXT_Full_ | .00 | 0.03 | -0.16 | .873 | .00 | 0.05 | -0.31 | .760 |
| AV_nocost-cs-_ | .00 | 0.25 | 1.42 | .155 | .00 | 0.20 | 1.10 | .270 |
| AV_nocost-cs+_ | .00 | 0.08 | 0.46 | .644 | .00 | 0.23 | 1.31 | .192 |
| GEN | .00 | -0.04 | -0.24 | .814 | .00 | -0.03 | -0.16 | .873 |
| **GAD-7** |  |  |  |  |  |  |  |  |
| Baseline | .46 | 0.63 | 21.21 | < .001*** | .48 | 0.71 | 22.09 | < .001*** |
| ACQ_csplus_ | .00 | -0.06 | -0.45 | .655 | 0.00 | -0.01 | -0.09 | .930 |
| ACQ_csmin_ | .00 | 0.21 | 1.48 | .140 | 0.00 | 0.23 | 1.42 | .155 |
| EXT_Early_ | .00 | 0.06 | -0.36 | .722 | .00 | -0.01 | 0.08 | .933 |
| EXT_Full_ | .00 | 0.07 | -0.43 | .665 | .00 | -0.01 | 0.06 | .950 |
| AV_nocost-csmin_ | .00 | 0.15 | 0.91 | .366 | .00 | 0.23 | 1.29 | .197 |
| AV_nocost-csplus_ | .00 | 0.08 | 0.47 | .640 | .01 | 0.36 | 2.00 | .046* |
| GEN | .00 | -0.19 | -1.23 | .218 | .00 | 0.14 | 0.84 | .401 |
| **DASS-S** |  |  |  |  |  |  |  |  |
| Baseline | .31 | 0.56 | 15.55 | < .001*** | .31 | 0.58 | 15.58 | < .001*** |
| ACQ_csplus_ | .00 | 0.08 | 0.48 | .630 | .00 | 0.12 | 0.67 | .506 |
| ACQ_csmin_ | .01 | 0.35 | 2.06 | .040* | .01 | 0.52 | 2.90 | .004** |
| EXT_Early_ | .00 | 0.08 | -0.43 | .665 | .00 | 0.14 | -0.73 | .463 |
| EXT_Full_ | .00 | 0.11 | -0.56 | .576 | .00 | 0.17 | -0.87 | .382 |
| AV_nocost-csmin_ | .00 | 0.17 | 0.85 | .396 | .00 | 0.16 | 0.77 | .441 |
| AV_nocost-cs+_ | .00 | 0.13 | 0.62 | .537 | .01 | 0.37 | 1.77 | .078 |
| GEN | .00 | -0.19 | -1.00 | .319 | .00 | -0.01 | -0.03 | .977 |
| **DASS-D** |  |  |  |  |  |  |  |  |
| Baseline | .30 | 0.55 | 15.12 | < .001*** | .32 | 0.59 | 15.65 | < .001*** |
| Acq_csplus_ | .00 | 0.04 | 0.21 | .833 | .00 | -0.06 | -0.34 | .732 |
| Acq_csmin_ | .00 | 0.32 | 1.80 | .073 | .01 | 0.53 | 2.87 | .004** |
| EXT_Early_ | .00 | 0.06 | -0.29 | .770 | .00 | 0.03 | -0.15 | .882 |
| EXT_Full_ | .00 | 0.09 | -0.49 | .622 | .00 | 0.05 | -0.23 | .815 |
| AV_nocost-csmin_ | .00 | 0.29 | 1.41 | .160 | .00 | 0.17 | 0.78 | .438 |
| AV_nocost-csplus_ | .00 | -0.12 | -0.57 | .571 | .00 | 0.18 | 0.83 | .407 |
| GEN | .00 | -0.11 | -0.59 | .557 | .00 | 0.12 | 0.62 | .532 |

*Note*. DASS-A, DASS-S, DASS-D = subscales of the Depression Anxiety Stress Scales; GAD = Generalized Anxiety Disorder seven item scale; ACQ_csplus_ = acquisition index CS+ trials based on expectancy ratings; ACQ_csmin_ = acquisition index CS- trials based on expectancy ratings; EXT_Early_ = early extinction index based on expectancy ratings; EXT_Full_ = full extinction index based on expectancy ratings; AV_nocost_csmin_ = proportion avoided CS- trials without cost, AV_nocost_csplus_ = proportion avoided CS+ trials without cost, GEN = generalization index based on expectancy ratings.

Baseline = baseline measurement of the corresponding questionnaire (Part 1); Part 2 = post-measurement in February; Part 3 = post-measurement in March

^a^ Grey values: compared to the Step 0 model (only intercept); white values: compared to the Step 1 model (indicated in grey).

*** *p* < .001; ** *p* < .01; * *p* < .05

**Table 2**

*Prediction of Anxiety, Stress, And Depression from Secondary Learning Indices*

|  | **Part 2** | | | | **Part 3** | | | |
| --- | --- | --- | --- | --- | --- | --- | --- | --- |
|  | **∆*R^2^*** | **β** | ***t*** | ***p*** | **∆*R^2^*** | **β** | ***t*** | ***p*** |
| **DASS-A** |  |  |  |  |  |  |  |  |
| Baseline | .36 | 0.61 | 17.55 | < .001*** | .34 | 0.60 | 16.60 | < .001*** |
| ACQ_Dist-csplus_ | .00 | 0.21 | 1.44 | .151 | .00 | 0.11 | 0.73 | .465 |
| ACQ_Dist-csmin_ | .00 | 0.08 | 0.55 | .583 | .00 | 0.10 | 0.65 | .518 |
| EXT_Dist-Early_ | .00 | 0.00 | 0.01 | .991 | .00 | -0.07 | 0.42 | .678 |
| EXT_Dist-Full_ | .00 | 0.22 | -1.38 | .168 | .00 | 0.04 | -0.22 | .827 |
| AV_cost-csplus_ | .01 | 0.42 | 2.00 | .047* | .00 | 0.06 | 0.29 | .772 |
| GEN_Dist_ | .00 | 0.11 | 0.65 | .518 | .00 | 0.07 | 0.42 | .677 |
| **GAD-7** |  |  |  |  |  |  |  |  |
| Baseline | .46 | 0.63 | 21.21 | < .001*** | .48 | 0.71 | 22.09 | < .001*** |
| ACQ_Dist-csplus_ | .00 | -0.04 | -0.29 | .772 | .00 | 0.05 | 0.34 | .735 |
| ACQ_Dist-csmin_ | .00 | 0.07 | 0.49 | .624 | .00 | 0.04 | 0.28 | .781 |
| EXT_Dist-Early_ | .00 | 0.00 | 0.01 | .995 | .00 | -0.02 | 0.11 | .916 |
| EXT_Dist-Full_ | .00 | 0.16 | -0.97 | .331 | .00 | 0.14 | -0.80 | .425 |
| AV_cost-csplus_ | .00 | -0.02 | -0.08 | .935 | .00 | -0.12 | -0.56 | .574 |
| GEN_Dist_ | .00 | 0.06 | 0.36 | .718 | .00 | 0.08 | 0.46 | .644 |
| **DASS-S** |  |  |  |  |  |  |  |  |
| Baseline | .31 | 0.56 | 15.55 | < .001*** | .31 | 0.58 | 15.58 | < .001*** |
| ACQ_Dist-csplus_ | .00 | 0.21 | 1.21 | .227 | 0.00 | 0.20 | 1.13 | .260 |
| ACQ_Dist-csmin_ | .00 | 0.27 | 1.55 | .122 | 0.00 | 0.18 | 1.02 | .309 |
| EXT_Dist-Early_ | .00 | -0.05 | 0.27 | .785 | .00 | -0.13 | 0.62 | .537 |
| EXT_Dist-Full_ | .00 | 0.08 | -0.43 | .670 | .00 | 0.01 | -0.05 | .961 |
| AV_cost-csplus_ | .00 | 0.07 | 0.31 | .760 | .00 | 0.18 | 0.71 | .476 |
| GEN_Dist_ | .00 | -0.06 | -0.29 | .769 | .00 | -0.09 | -0.46 | .644 |
| **DASS-D** |  |  |  |  |  |  |  |  |
| Baseline | .30 | 0.55 | 15.12 | < .001*** | .32 | 0.59 | 15.65 | < .001*** |
| ACQ_Dist-csplus_ | .00 | 0.07 | 0.42 | .672 | .00 | 0.08 | 0.41 | .679 |
| ACQ_Dist-csmin_ | .00 | 0.17 | 0.95 | .345 | .00 | 0.19 | 1.02 | .306 |
| EXT_Dist-Early_ | .00 | 0.09 | -0.45 | .656 | .00 | -0.07 | 0.31 | .756 |
| EXT_Dist-Full_ | .00 | 0.09 | -0.44 | .657 | .00 | 0.01 | -0.03 | .976 |
| AV_cost-csplus_ | .01 | 0.40 | 1.64 | .102 | .00 | 0.30 | 1.17 | .243 |
| GEN_Dist_ | .01 | 0.08 | 1.56 | .120 | .01 | 0.07 | 1.30 | .195 |

*Note*. DASS-A, DASS-S, DASS-D = subscales of the Depression Anxiety Stress Scales; GAD = Generalized Anxiety Disorder seven item scale; ACQ_Dist-csplus_ = acquisition index CS+ trials based on distress ratings; ACQ_Dist-csmin_ = acquisition index CS- trials based on distress ratings; EXT_Dist-Early_ = early extinction index based on distress ratings; EXT_Dist-Full_ = full extinction index based on distress ratings; AV_cost_csplus_ = proportion avoided CS+ trials without cost, GEN_Dist_ = generalization index based on distress ratings. Baseline = baseline measurement of the corresponding questionnaire (Part 1), Part 2 = post-measurement in February; Part 3 = post-measurement in March

^a^ Grey values: compared to the Step 0 model (only intercept); white values: compared to the Step 1 model (indicated in grey).

*** *p* < .001; ** *p* < .01; * *p* < .05

**Table 2**

*Predictions from interactions: primary indices*

|  | **Part 2** | | | | **Part 3** | | | |
| --- | --- | --- | --- | --- | --- | --- | --- | --- |
|  | **∆*R^2^*** | **β** | ***t*** | ***p*** | **∆*R^2^*** | **β** | ***t*** | ***p*** |
| **DASS-A** |  |  |  |  |  |  |  |  |
| ACQ_csplus_ x ACQ_csmin_ | .01 | 0.33 | 2.06 | .040* | .00 | 0.09 | 0.56 | .575 |
| ACQ_csplus_ x EXT_Early_ | .00 | -0.14 | -0.90 | .371 | .00 | -0.28 | -1.73 | .084 |
| ACQ_csplus_ x EXT_Full_ | .00 | -0.18 | -1.22 | .223 | .00 | -0.25 | -1.60 | .110 |
| ACQ_csplus_ x AV_nocost-cs+_ | .01 | -0.38 | -2.28 | .023* | .01 | -0.37 | -2.09 | .038* |
| ACQ_csplus_ x AV_nocost-cs-_ | .00 | 0.06 | 0.28 | .778 | .00 | 0.28 | 1.34 | .181 |
| ACQ_csplus_ x GEN | .00 | -0.14 | -0.87 | .386 | .00 | 0.01 | 0.09 | .926 |
| ACQ_csmin_ x EXT_Early_ | .00 | -0.06 | -0.36 | .722 | .00 | -0.09 | -0.54 | .587 |
| ACQ_csmin_ x EXT_Full_ | .00 | -0.07 | -0.41 | .679 | .00 | -0.09 | -0.54 | .587 |
| ACQ_csmin_ x AV_nocost-cs+_ | .00 | -0.14 | -0.72 | .471 | .00 | -0.22 | -1.11 | .269 |
| ACQ_csmin_ x AV_nocost-cs-_ | .00 | 0.00 | 0.00 | .997 | .00 | -0.17 | -0.81 | .420 |
| ACQ_csmin_ x GEN | .00 | -0.02 | -0.10 | .919 | .00 | -0.07 | -0.41 | .680 |
| EXT_Early_ x EXT_Full_ | .00 | -0.12 | -0.90 | .371 | .00 | -0.15 | -1.11 | .269 |
| EXT_Early_ x AV_nocost-cs+_ | .00 | -0.04 | -0.22 | .825 | .00 | -0.02 | -0.12 | .904 |
| EXT_Early_ x AV_nocost-cs-_ | .00 | -0.02 | -0.07 | .941 | .00 | 0.32 | 1.45 | .147 |
| EXT_Early_ x GEN | .00 | 0.13 | 0.74 | .459 | .00 | 0.13 | 0.73 | .467 |
| EXT_Full_ x AV_nocost-cs+_ | .00 | 0.20 | 1.04 | .298 | .00 | 0.20 | 1.04 | .300 |
| EXT_Full_ x AV_nocost-cs-_ | .00 | -0.06 | -0.30 | .764 | .00 | 0.19 | 0.89 | .373 |
| EXT_Full_ x GEN | .00 | 0.10 | 0.53 | .595 | .00 | 0.02 | 0.11 | .912 |
| AV_nocost-cs+_ x AV_nocost-cs-_ | .00 | -0.06 | -0.26 | .798 | .00 | 0.06 | 0.24 | .813 |
| AV_nocost-cs+_ x GEN | .00 | 0.11 | 0.56 | .576 | .00 | 0.19 | 1.00 | .318 |
| AV_nocost-cs-_ x GEN | .00 | 0.20 | 0.99 | .322 | .00 | 0.06 | 0.32 | .750 |
| **DASS-S** |  |  |  |  |  |  |  |  |
| ACQ_csplus_ x ACQ_csmin_ | .01 | 0.50 | 2.71 | .007** | .00 | 0.09 | 0.45 | .654 |
| ACQ_csplus_ x EXT_Early_ | .01 | -0.39 | -2.13 | .033* | .01 | -0.44 | -2.35 | .019* |
| ACQ_csplus_ x EXT_Full_ | .01 | -0.36 | -1.98 | .048* | .01 | -0.35 | -1.86 | .064 |
| ACQ_csplus_ x AV_nocost-cs+_ | .00 | -0.31 | -1.62 | .105 | .00 | -0.17 | -0.83 | .407 |
| ACQ_csplus_ x AV_nocost-cs-_ | .01 | 0.43 | 1.90 | .059 | .00 | 0.36 | 1.51 | .132 |
| ACQ_csplus_ x GEN | .00 | 0.01 | 0.06 | .953 | .00 | 0.15 | 0.81 | .417 |
| ACQ_csmin_ x EXT_Early_ | .00 | -0.06 | -0.31 | .754 | .01 | -0.38 | -1.94 | .053 |
| ACQ_csmin_ x EXT_Full_ | .00 | -0.14 | -0.70 | .485 | .01 | -0.43 | -2.17 | .031* |
| ACQ_csmin_ x AV_nocost-cs+_ | .01 | -0.44 | -2.01 | .045* | .00 | -0.21 | -0.93 | .353 |
| ACQ_csmin_ x AV_nocost-cs-_ | .00 | -0.27 | -1.18 | .240 | .00 | -0.17 | -0.70 | .485 |
| ACQ_csmin_ x GEN | .00 | -0.09 | -0.45 | .652 | .00 | -0.02 | -0.09 | .928 |
| EXT_Early_ x EXT_Full_ | .00 | 0.10 | 0.62 | .538 | .00 | -0.10 | -0.63 | .529 |
| EXT_Early_ x AV_nocost-cs+_ | .00 | -0.14 | -0.60 | .549 | .00 | -0.04 | -0.15 | .878 |
| EXT_Early_ x AV_nocost-cs-_ | .00 | 0.00 | 0.01 | .990 | .00 | 0.22 | 0.81 | .420 |
| EXT_Early_ x GEN | .00 | 0.30 | 1.40 | .163 | .00 | -0.04 | -0.17 | .862 |
| EXT_Full_ x AV_nocost-cs+_ | .00 | 0.20 | 0.88 | .379 | .00 | 0.13 | 0.55 | .580 |
| EXT_Full_ x AV_nocost-cs-_ | .00 | -0.02 | -0.09 | .929 | .00 | 0.09 | 0.33 | .743 |
| EXT_Full_ x GEN | .00 | 0.26 | 1.19 | .234 | .00 | -0.05 | -0.25 | .804 |
| AV_nocost-cs+_ x AV_nocost-cs-_ | .00 | -0.19 | -0.70 | .487 | .00 | 0.16 | 0.56 | .579 |
| AV_nocost-cs+_ x GEN | .00 | 0.06 | 0.25 | .801 | .01 | 0.47 | 2.15 | .032* |
| AV_nocost-cs-_ x GEN | .00 | -0.30 | -1.28 | .200 | .00 | -0.05 | -0.22 | .828 |
| **DASS-D** |  |  |  |  |  |  |  |  |
| ACQ_csplus_ x ACQ_csmin_ | .00 | 0.25 | 1.30 | .194 | 0.00 | 0.07 | 0.36 | .718 |
| ACQ_csplus_ x EXT_Early_ | .00 | -0.20 | -1.09 | .274 | 0.00 | -0.19 | -0.95 | .342 |
| ACQ_csplus_ x EXT_Full_ | .00 | -0.19 | -1.06 | .291 | 0.00 | -0.10 | -0.48 | .629 |
| ACQ_csplus_ x AV_nocost-cs+_ | .00 | -0.16 | -0.80 | .426 | 0.00 | 0.03 | 0.13 | .900 |
| ACQ_csplus_ x AV_nocost-cs-_ | .00 | 0.37 | 1.55 | .122 | 0.00 | 0.35 | 1.42 | .156 |
| ACQ_csplus_ x GEN | .00 | -0.10 | -0.51 | .613 | 0.00 | 0.07 | 0.36 | .722 |
| ACQ_csmin_ x EXT_Early_ | .00 | 0.00 | -0.01 | .991 | 0.00 | -0.19 | -0.90 | .369 |
| ACQ_csmin_ x EXT_Full_ | .00 | -0.08 | -0.44 | .662 | 0.00 | -0.24 | -1.15 | .252 |
| ACQ_csmin_ x AV_nocost-cs+_ | .00 | -0.19 | -0.85 | .396 | 0.00 | -0.21 | -0.90 | .366 |
| ACQ_csmin_ x AV_nocost-cs-_ | .00 | 0.26 | 1.09 | .278 | 0.00 | 0.16 | 0.64 | .521 |
| ACQ_csmin_ x GEN | .00 | 0.21 | 1.02 | .306 | 0.00 | -0.10 | -0.46 | .644 |
| EXT_Early_ x EXT_Full_ | .00 | 0.05 | 0.31 | .759 | .00 | 0.01 | 0.04 | .969 |
| EXT_Early_ x AV_nocost-cs+_ | .00 | 0.30 | 1.26 | .209 | .00 | -0.15 | -0.58 | .562 |
| EXT_Early_ x AV_nocost-cs-_ | .00 | 0.24 | 0.91 | .366 | .00 | 0.20 | 0.72 | .472 |
| EXT_Early_ x GEN | .00 | 0.23 | 1.08 | .282 | .00 | 0.05 | 0.21 | .831 |
| EXT_Full_ x AV_nocost-cs+_ | .00 | 0.34 | 1.48 | .140 | .00 | -0.17 | -0.71 | .479 |
| EXT_Full_ x AV_nocost-cs-_ | .00 | 0.28 | 1.08 | .280 | .00 | 0.14 | 0.50 | .616 |
| EXT_Full_ x GEN | .00 | 0.26 | 1.22 | .225 | .00 | 0.00 | 0.00 | .998 |
| AV_nocost-cs+_ x AV_nocost-cs-_ | .00 | -0.30 | -1.06 | .292 | .00 | -0.19 | -0.66 | .510 |
| AV_nocost-cs+_ x GEN | .00 | 0.24 | 1.03 | .303 | .01 | 0.48 | 2.12 | .035* |
| AV_nocost-cs-_ x GEN | .00 | -0.19 | -0.79 | .432 | .00 | -0.04 | -0.18 | .858 |
| **GAD-7** |  |  |  |  |  |  |  |  |
| ACQ_csplus_ x ACQ_csmin_ | .00 | 0.15 | 0.93 | .355 | .00 | -0.03 | -0.16 | .870 |
| ACQ_csplus_ x EXT_Early_ | .01 | -0.36 | -2.35 | .019* | .00 | -0.25 | -1.50 | .134 |
| ACQ_csplus_ x EXT_Full_ | .01 | -0.41 | -2.78 | .006** | .00 | -0.30 | -1.81 | .071 |
| ACQ_csplus_ x AV_nocost-cs+_ | .00 | -0.22 | -1.37 | .171 | .00 | -0.26 | -1.47 | .142 |
| ACQ_csplus_ x AV_nocost-cs-_ | .01 | 0.54 | 2.90 | .004** | .00 | 0.25 | 1.20 | .232 |
| ACQ_csplus_ x GEN | .00 | -0.02 | -0.16 | .874 | .00 | 0.06 | 0.34 | .735 |
| ACQ_csmin_ x EXT_Early_ | .00 | -0.09 | -0.57 | .570 | .00 | -0.16 | -0.90 | .366 |
| ACQ_csmin_ x EXT_Full_ | .00 | -0.18 | -1.11 | .266 | .00 | -0.25 | -1.40 | .161 |
| ACQ_csmin_ x AV_nocost-cs+_ | .00 | -0.17 | -0.95 | .342 | .00 | -0.01 | -0.03 | .979 |
| ACQ_csmin_ x AV_nocost-cs-_ | .00 | -0.07 | -0.39 | .699 | .00 | 0.00 | 0.01 | .995 |
| ACQ_csmin_ x GEN | .00 | -0.03 | -0.17 | .861 | .00 | -0.08 | -0.44 | .657 |
| EXT_Early_ x EXT_Full_ | .00 | 0.12 | 0.87 | .387 | .00 | -0.05 | -0.33 | .742 |
| EXT_Early_ x AV_nocost-cs+_ | .00 | 0.09 | 0.46 | .645 | .00 | -0.07 | -0.32 | .752 |
| EXT_Early_ x AV_nocost-cs-_ | .00 | 0.00 | 0.00 | .997 | .00 | -0.07 | -0.33 | .745 |
| EXT_Early_ x GEN | .00 | 0.10 | 0.59 | .555 | .00 | 0.00 | 0.02 | .984 |
| EXT_Full_ x AV_nocost-cs+_ | .00 | 0.21 | 1.16 | .245 | .00 | 0.12 | 0.60 | .548 |
| EXT_Full_ x AV_nocost-cs-_ | .00 | -0.01 | -0.04 | .971 | .00 | -0.09 | -0.41 | .684 |
| EXT_Full_ x GEN | .00 | 0.12 | 0.68 | .498 | .00 | 0.00 | -0.01 | .996 |
| AV_nocost-cs+_ x AV_nocost-cs-_ | .00 | 0.22 | 0.98 | .325 | .00 | 0.18 | 0.73 | .463 |
| AV_nocost-cs+_ x GEN | .00 | -0.10 | -0.57 | .570 | .00 | 0.34 | 1.79 | .074 |
| AV_nocost-cs-_ x GEN | .00 | 0.06 | 0.30 | .768 | .00 | 0.25 | 1.19 | .236 |

*Note*. DASS-A, DASS-S, DASS-D = subscales of the Depression Anxiety Stress Scales; GAD = Generalized Anxiety Disorder seven item scale; ACQ_csplus_ = acquisition index CS+ trials based on expectancy ratings; ACQ_csmin_ = acquisition index CS- trials based on expectancy ratings; EXT_Early_ = early extinction index based on expectancy ratings; EXT_Full_ = full extinction index based on expectancy ratings; AV_nocost_csmin_ = proportion avoided CS- trials without cost, AV_nocost_csplus_ = proportion avoided CS+ trials without cost, GEN = generalization index based on expectancy ratings; Part 2 = post-measurement in February; Part 3 = post-measurement in March

^a^ *R²* compared to a model containing only baseline questionnaire measures and both predictors (no interactions).

*** *p* < .001; ** *p* < .01; * *p* < .05

## Supplementary Material 6 - Predicting the Impact of Failing Exams (Full Tables)

**Table 1**

*Prediction of Changes In Anxiety, Stress, And Depression, in Interaction with Disappointing Failed Exams (Primary Indices)*

|  | **Part 2** | | | | **Part 3** | | | |
| --- | --- | --- | --- | --- | --- | --- | --- | --- |
|  | **∆*R^2^*** | **β** | ***t*** | ***p*** | **∆*R^2^*** | **β** | ***t*** | ***p*** |
| **DASS-A** |  |  |  |  |  |  |  |  |
| Failed | .01 | 0.78 | 2.55 | .011* | .01 | 0.67 | 2.16 | .031* |
| Failed x ACQ_csplus_ | .00 | 0.24 | 0.78 | .437 | .00 | -0.04 | -0.13 | .893 |
| Failed x ACQ_csmin_ | .00 | -0.18 | -0.58 | .561 | .00 | -0.19 | -0.60 | .547 |
| Failed x EXT_Early_ | .00 | -0.15 | -0.45 | .656 | .00 | -0.17 | -0.50 | .616 |
| Failed x EXT_Full_ | .00 | 0.04 | 0.13 | .893 | .00 | 0.13 | 0.40 | .691 |
| Failed x AV_nocost-cs+_ | .00 | 0.18 | 0.49 | .623 | .00 | 0.22 | 0.61 | .542 |
| Failed x AV_nocost-cs-_ | .00 | 0.45 | 1.20 | .229 | .00 | 0.13 | 0.35 | .728 |
| Failed x GEN | .00 | 0.38 | 1.12 | .261 | .00 | 0.24 | 0.72 | .469 |
| **DASS-S** |  |  |  |  |  |  |  |  |
| Failed | .01 | 1.13 | 3.23 | .001** | .01 | 0.73 | 2.04 | .042* |
| Failed x ACQ_csplus_ | .00 | -0.15 | -0.44 | .663 | .00 | -0.39 | -1.06 | .291 |
| Failed x ACQ_csmin_ | .00 | -0.06 | -0.18 | .858 | .00 | 0.07 | 0.19 | .847 |
| Failed x EXT_Early_ | .00 | -0.18 | -0.47 | .635 | .00 | 0.26 | 0.66 | .510 |
| Failed x EXT_Full_ | .00 | -0.08 | -0.20 | .841 | .00 | 0.31 | 0.79 | .431 |
| Failed x AV_nocost-cs+_ | .00 | -0.26 | -0.63 | .530 | .00 | -0.20 | -0.47 | .637 |
| Failed x AV_nocost-cs-_ | .00 | 0.46 | 1.06 | .288 | .00 | 0.25 | 0.57 | .571 |
| Failed x GEN | .00 | -0.02 | -0.05 | .958 | .00 | -0.23 | -0.58 | .562 |
| **DASS-D** |  |  |  |  |  |  |  |  |
| Failed | .01 | 0.85 | 2.37 | .018* | .00 | 0.71 | 1.91 | .057 |
| Failed x ACQ_csplus_ | .00 | 0.02 | 0.06 | .949 | .00 | -0.25 | -0.66 | .510 |
| Failed x ACQ_csmin_ | .00 | -0.42 | -1.19 | .234 | .00 | -0.17 | -0.45 | .653 |
| Failed x EXT_Early_ | .00 | -0.02 | -0.06 | .955 | .00 | -0.31 | -0.73 | .465 |
| Failed x EXT_Full_ | .00 | -0.10 | -0.27 | .788 | .00 | -0.39 | -0.94 | .349 |
| Failed x AV_nocost-cs+_ | .00 | 0.38 | 0.87 | .384 | .00 | 0.22 | 0.49 | .623 |
| Failed x AV_nocost-cs-_ | .00 | 0.27 | 0.60 | .549 | .00 | 0.51 | 1.11 | .266 |
| Failed x GEN | .00 | 0.14 | 0.34 | .734 | .00 | -0.32 | -0.79 | .432 |
| **GAD-7** |  |  |  |  |  |  |  |  |
| Failed | .01 | 1.03 | 3.50 | <.001*** | .01 | 0.79 | 2.45 | .015* |
| Failed x ACQ_csplus_ | .00 | -0.22 | -0.73 | .466 | .01 | -0.78 | -2.36 | .019* |
| Failed x ACQ_csmin_ | .00 | -0.03 | -0.12 | .907 | .00 | 0.17 | 0.51 | .613 |
| Failed x EXT_Early_ | .00 | -0.22 | -0.70 | .485 | .00 | 0.03 | 0.07 | .941 |
| Failed x EXT_Full_ | .00 | 0.04 | 0.11 | .912 | .00 | 0.24 | 0.69 | .489 |
| Failed x AV_nocost-cs+_ | .00 | 0.60 | 1.76 | .080 | .00 | 0.44 | 1.21 | .229 |
| Failed x AV_nocost-cs-_ | .00 | 0.61 | 1.74 | .082 | .00 | 0.27 | 0.71 | .477 |
| Failed x GEN | .00 | 0.35 | 1.09 | .276 | .00 | 0.36 | 1.03 | .303 |
| **ATS** |  |  |  |  |  |  |  |  |
| ACQ_csplus_ | .00 | -0.01 | -0.19 | .852 | .00 | -0.02 | -0.46 | .649 |
| ACQ_csmin_ | .03 | 0.16 | 3.14 | .002** | .02 | 0.15 | 2.69 | .007** |
| EXT_Early_ | .00 | -0.06 | -0.92 | .356 | .00 | -0.05 | -0.80 | .426 |
| EXT_Full_ | .00 | -0.02 | -0.33 | .742 | .00 | -0.01 | -0.13 | .896 |
| AV_nocost-cs+_ | .01 | 0.07 | 1.18 | .239 | .00 | 0.00 | 0.07 | .946 |
| AV_nocost-cs-_ | .01 | 0.07 | 1.14 | .257 | .00 | 0.01 | 0.17 | .869 |
| GEN | .00 | 0.02 | 0.38 | .701 | .00 | 0.05 | 0.79 | .430 |

*Note*. DASS-A, DASS-S, DASS-D = subscales of the Depression Anxiety Stress Scales; GAD = Generalized Anxiety Disorder seven item scale; Failed = dummy variable receival of disappointing failed exam results; ACQ_csplus_ = acquisition index CS+ trials based on expectancy ratings; ACQ_csmin_ = acquisition index CS- trials based on expectancy ratings; EXT_Early_ = early extinction index based on expectancy ratings; EXT_Full_ = full extinction index based on expectancy ratings; AV_nocost_csmin_ = proportion avoided CS- trials without cost, AV_nocost_csplus_ = proportion avoided CS+ trials without cost, GEN = generalization index based on expectancy ratings; Part 2 = post-measurement in February; Part 3 = post-measurement in March

^a^ Grey values: compared to the Step 0 model (only intercept); white values: compared to the Step 1 model (indicated in grey).

*** *p* < .001; ** *p* < .01; * *p* < .05

**Table 2**

*Prediction of Changes In Anxiety, Stress, And Depression, in Interaction with Disappointing Failed Exams (Secondary Indices)*

|  | **Part 2** | | | | **Part 3** | | | |
| --- | --- | --- | --- | --- | --- | --- | --- | --- |
|  | **∆*R^2^*** | **β** | ***t*** | ***p*** | **∆*R^2^*** | **β** | ***t*** | ***p*** |
| **DASS-A** |  |  |  |  |  |  |  |  |
| Failed x ACQ_Dist-csplus_ | .00 | 0.57 | 1.89 | .060 | .00 | 0.38 | 1.21 | .229 |
| Failed x ACQ_Dist-csmin_ | .00 | 0.18 | 0.60 | .551 | .00 | 0.18 | 0.57 | .568 |
| Failed x EXT_Dist-Early_ | .00 | 0.00 | 0.01 | .989 | .00 | -0.37 | -1.06 | .290 |
| Failed x EXT_Dist-Full_ | .00 | -0.20 | -0.59 | .553 | .00 | -0.28 | -0.83 | .407 |
| Failed x AV_cost-csplus_ | .01 | 0.81 | 1.87 | .063 | .00 | 0.18 | 0.42 | .678 |
| Failed x GEN_Dist_ | .00 | 0.24 | 0.67 | .504 | .00 | 0.41 | 1.21 | .226 |
| **DASS-S** |  |  |  |  |  |  |  |  |
| Failed x ACQ_Dist-csplus_ | .00 | 0.17 | 0.47 | .635 | .00 | -0.26 | -0.72 | .471 |
| Failed x ACQ_Dist-csmin_ | .00 | 0.24 | 0.67 | .505 | .00 | 0.00 | -0.01 | .992 |
| Failed x EXT_Dist-Early_ | .00 | -0.24 | -0.59 | .553 | .00 | 0.30 | 0.73 | .467 |
| Failed x EXT_Dist-Full_ | .00 | -0.09 | -0.23 | .816 | .00 | 0.55 | 1.36 | .175 |
| Failed x AV_cost-csplus_ | .00 | 0.39 | 0.79 | .428 | .00 | -0.44 | -0.88 | .382 |
| Failed x GEN_Dist_ | .00 | 0.53 | 1.31 | .191 | .00 | 0.68 | 1.70 | .089 |
| **DASS-D** |  |  |  |  |  |  |  |  |
| Failed x ACQ_Dist-csplus_ | .00 | -0.04 | -0.11 | .910 | .00 | 0.24 | 0.65 | .518 |
| Failed x ACQ_Dist-csmin_ | .00 | -0.26 | -0.70 | .482 | .00 | 0.20 | 0.52 | .600 |
| Failed x EXT_Dist-Early_ | .00 | 0.54 | 1.34 | .180 | .00 | 0.03 | 0.07 | .944 |
| Failed x EXT_Dist-Full_ | .00 | 0.08 | 0.21 | .832 | .00 | -0.05 | -0.13 | .898 |
| Failed x AV_cost-csplus_ | .02 | 1.20 | 2.43 | .016* | .00 | -0.30 | -0.59 | .554 |
| Failed x GEN_Dist_ | .00 | 0.25 | 0.61 | .541 | .00 | 0.33 | 0.79 | .428 |
| **GAD-7** |  |  |  |  |  |  |  |  |
| Failed x ACQ_Dist-csplus_ | .00 | 0.06 | 0.19 | .847 | .00 | -0.26 | -0.79 | .432 |
| Failed x ACQ_Dist-csmin_ | .00 | 0.23 | 0.78 | .438 | .00 | 0.11 | 0.33 | .740 |
| Failed x EXT_Dist-Early_ | .00 | 0.20 | 0.59 | .556 | .00 | 0.63 | 1.73 | .084 |
| Failed x EXT_Dist-Full_ | .00 | 0.07 | 0.21 | .834 | .01 | 0.74 | 2.12 | .035* |
| Failed x AV_cost-csplus_ | .00 | 0.27 | 0.71 | .477 | .00 | -0.17 | -0.38 | .704 |
| Failed x GEN_Dist_ | .00 | 0.41 | 1.26 | .210 | .00 | 0.49 | 1.39 | .164 |
| **ATS** |  |  |  |  |  |  |  |  |
| ACQ_Dist-csplus_ | .00 | 0.03 | 0.57 | .568 | .00 | 0.05 | 1.02 | .309 |
| ACQ_Dist-csmin_ | .02 | 0.12 | 2.30 | .022* | .01 | 0.11 | 2.00 | .046* |
| EXT_Dist-Early_ | .00 | 0.02 | 0.35 | .723 | .00 | 0.00 | -0.02 | .980 |
| EXT_Dist-Full_ | .00 | 0.04 | 0.64 | .525 | .00 | 0.01 | 0.09 | .930 |
| AV_cost-csplus_ | .00 | -0.01 | -0.12 | .902 | .01 | -0.12 | -1.49 | .138 |
| GEN_Dist_ | .01 | 0.08 | 1.56 | .120 | .01 | 0.07 | 1.30 | .195 |

*Note*. DASS-A, DASS-S, DASS-D = subscales of the Depression Anxiety Stress Scales; GAD = Generalized Anxiety Disorder seven item scale; Failed = dummy variable receival of disappointing failed exam results; ACQ_Dist-csplus_ = acquisition index CS+ trials based on distress ratings; ACQ_Dist-csmin_ = acquisition index CS- trials based on distress ratings; EXT_Dist-Early_ = early extinction index based on distress ratings; EXT_Dist-Full_ = full extinction index based on distress ratings; AV_cost_csplus_ = proportion avoided CS+ trials without cost, GEN = generalization index based on distress ratings; Part 2 = post-measurement in February; Part 3 = post-measurement in March

^a^ *R²* compared to the model indicated in grey (Table 1)

*** *p* < .001; ** *p* < .01; * *p* < .05

## Supplementary Material 7 – Impact of Failing Exams: Trajectories (Growth Mixture Modeling)

**Table 1**

*Model Fit Statistics of the Single-Group Linear, Logarithmic, Quadratic, and Cubic Growth Models*

|  | **BIC** | **AIC** |
| --- | --- | --- |
| Linear | 17017 | 17000 |
| Logarithmic | 16973 | 16956 |
| **Quadratic** | **16971** | **16948** |
| Cubic | 16977 | 16949 |

*Note*. BIC = Bayesian Information Criterion; AIC = Akaike Information Criterion

**Table 2**

*Model Fit Statistics of the Growth Mixture Models (GMM)*

|  | ***N_classes_*** | **npm** | **loglik** | **BIC** | **AIC** | **Class-sizes** |
| --- | --- | --- | --- | --- | --- | --- |
| Fixed intercept + slope (LCGA) | 1 | 4 | -8469.85 | 16961.19 | 16947.69 | 100 |
|  | 2 | 7 | -7742.01 | 15521.64 | 15498.01 | 40, 60 |
|  | 3 | 10 | -7435.35 | 14924.45 | 14890.70 | 21, 46, 33 |
|  | 4 | 13 | -7340.17 | 14750.21 | 14706.33 | 42, 19, 32, 7 |
|  | 5 | 16 | -7257.07 | 14600.15 | 14546.15 | 17, 23, 7, 25, 29 |
|  | 6 | 19 | -7182.19 | 14466.51 | 14402.38 | 14, 16, 17, 7, 29, 18 |
| Random intercept | 1 | 5 | -7363.47 | 14753.81 | 14736.93 | 100 |
|  | 2 | 9 | -7153.17 | 14354.73 | 14324.35 | 38, 63 |
|  | 3 | 13 | -7089.11 | 14248.1 | 14204.22 | 17, 56, 27 |
|  | 4 | 17 | -7063.71 | 14218.8 | 14161.42 | 32, 28, 11, 29 |
|  | 5 | 21 | -7049.88 | 14212.64 | 14141.76 | 28, 10, 7, 25, 29 |
|  | 6 | 25 | -7041.61 | 14217.6 | 14133.22 | 31, 22, 17, 6, 10, 15 |
| Random intercept + slope | 1 | 7 | -7107.15 | 14251.92 | 14228.3 | 100 |
|  | 2 | 11 | -7069.08 | 14197.29 | 14160.16 | 75, 25 |
|  | **3** | **15** | **-7051.62** | **14183.87** | **14133.24** | **18, 18, 64** |
|  | 4 | 19 | -7047.42 | 14196.97 | 14132.84 | 30, 28, 16, 26 |
|  | 5 | 23 | -7041.06 | 14205.75 | 14128.12 | 16, 16, 20, 31, 17 |
|  | 6 | 27 | -7037.32 | 14219.78 | 14128.65 | 21, 14, 15, 15, 23, 12 |

*Note. N_classes_* = number of subgroups, npm = number of free parameters, loglik = log likelihood, BIC = Bayesian Information Criterion, AIC = Akaike Information Criterion, Class-sizes = percentage of participants in each subgroup

**Figure 1**

*Comparison Number of Classes for the Random Intercept and Slope Models
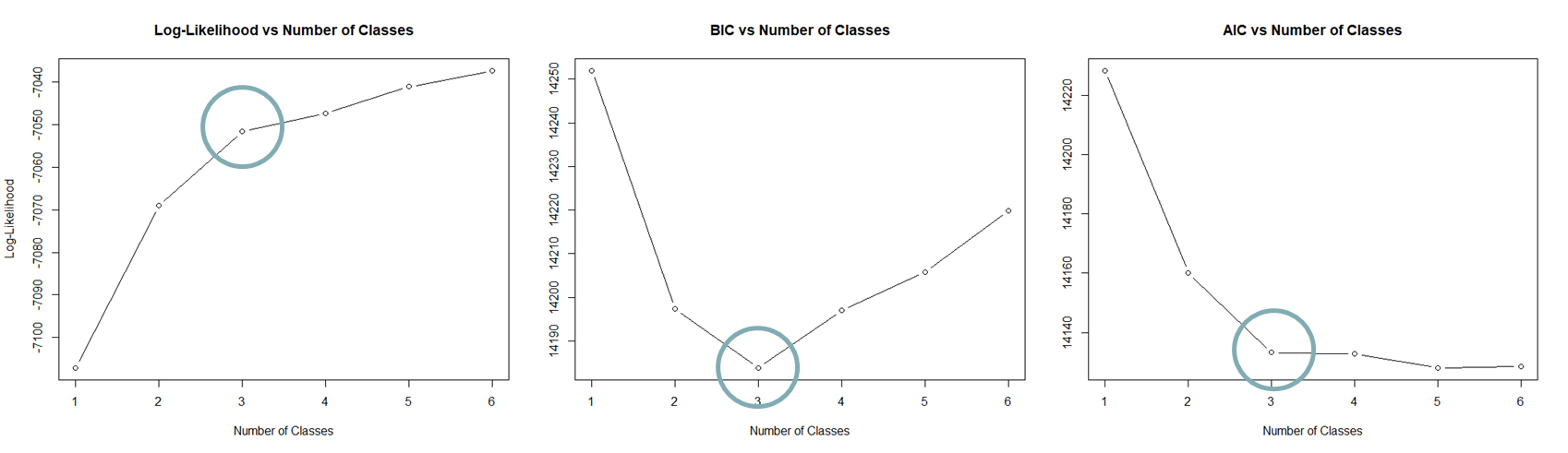
*

## Supplementary Material 8 - Predicting the Impact of Negative Life Events (Full Tables)

**Table 1**

*Prediction of Changes in Anxiety, Stress, and Depression, in Interaction with the Number of Negative Life Events (Primary Indices)*

|  | **Part 2** | | | | **Part 3** | | | |
| --- | --- | --- | --- | --- | --- | --- | --- | --- |
|  | **∆*R^2^*** | **β** | ***t*** | ***p*** | **∆*R^2^*** | **β** | ***t*** | ***p*** |
| **DASS-A** |  |  |  |  |  |  |  |  |
| NLE_N_ | .02 | 0.24 | 3.40 | <.001*** | 0.03 | 0.28 | 4.04 | <.001*** |
| NLE_N_ x ACQ_csplus_ | .01 | 0.20 | 2.68 | .008** | 0.00 | 0.05 | 0.68 | .497 |
| NLE_N_ x ACQ_csmin_ | .00 | 0.02 | 0.32 | .745 | 0.00 | 0.07 | 0.97 | .335 |
| NLE_N_ x EXT_Early_ | .00 | -0.06 | -0.90 | .369 | 0.00 | -0.06 | -0.89 | .373 |
| NLE_N_ x EXT_Full_ | .00 | -0.05 | -0.66 | .507 | 0.00 | -0.03 | -0.49 | .623 |
| NLE_N_ x AV_nocost-cs+_ | .00 | 0.10 | 1.23 | .218 | 0.00 | 0.08 | 1.09 | .278 |
| NLE_N_ x AV_nocost-cs-_ | .00 | 0.09 | 0.96 | .340 | 0.00 | 0.10 | 1.11 | .267 |
| NLE_N_ x GEN | .00 | -0.09 | -1.05 | .293 | 0.01 | -0.15 | -1.87 | .062 |
| **DASS-S** |  |  |  |  |  |  |  |  |
| NLE_N_ | .06 | 0.45 | 5.83 | <.001*** | 0.05 | 0.42 | 5.20 | <.001*** |
| NLE_N_ x ACQ_csplus_ | .02 | 0.24 | 3.00 | .003** | 0.01 | 0.16 | 1.84 | .066 |
| NLE_N_ x ACQ_csmin_ | .00 | 0.00 | -0.02 | .981 | 0.00 | 0.09 | 0.99 | .324 |
| NLE_N_ x EXT_Early_ | .00 | -0.02 | -0.20 | .843 | 0.00 | -0.01 | -0.07 | .948 |
| NLE_N_ x EXT_Full_ | .00 | -0.03 | -0.35 | .728 | 0.00 | 0.00 | 0.00 | .998 |
| NLE_N_ x AV_nocost-cs+_ | .01 | 0.20 | 2.34 | .020* | 0.01 | 0.15 | 1.71 | .089 |
| NLE_N_ x AV_nocost-cs-_ | .00 | 0.00 | 0.04 | .968 | 0.00 | 0.08 | 0.73 | .466 |
| NLE_N_ x GEN | .00 | -0.09 | -1.04 | .298 | 0.02 | -0.26 | -2.82 | .005** |
| **DASS-D** |  |  |  |  |  |  |  |  |
| NLE_N_ | .05 | 0.40 | 5.13 | <.001*** | 0.03 | 0.35 | 4.08 | <.001*** |
| NLE_N_ x ACQ_csplus_ | .01 | 0.17 | 2.03 | .043* | 0.00 | 0.10 | 1.12 | .265 |
| NLE_N_ x ACQ_csmin_ | .00 | -0.03 | -0.31 | .756 | 0.00 | -0.01 | -0.13 | .896 |
| NLE_N_ x EXT_Early_ | .00 | 0.03 | 0.36 | .722 | 0.00 | -0.01 | -0.07 | .944 |
| NLE_N_ x EXT_Full_ | .00 | 0.01 | 0.18 | .859 | 0.00 | -0.02 | -0.26 | .793 |
| NLE_N_ x AV_nocost-cs+_ | .01 | 0.15 | 1.77 | .078 | 0.01 | 0.21 | 2.23 | .026* |
| NLE_N_ x AV_nocost-cs-_ | .00 | 0.03 | 0.25 | .803 | 0.01 | 0.19 | 1.77 | .078 |
| NLE_N_ x GEN | .00 | -0.11 | -1.21 | .228 | 0.00 | -0.13 | -1.33 | .184 |
| **GAD-7** |  |  |  |  |  |  |  |  |
| NLE_N_ | .03 | 0.33 | 5.12 | <.001*** | 0.03 | 0.33 | 4.37 | <.001*** |
| NLE_N_ x ACQ_csplus_ | .00 | 0.11 | 1.73 | .084 | 0.00 | 0.01 | 0.08 | .935 |
| NLE_N_ x ACQ_csmin_ | .00 | 0.02 | 0.26 | .796 | 0.00 | 0.10 | 1.22 | .222 |
| NLE_N_ x EXT_Early_ | .00 | -0.04 | -0.69 | .492 | 0.00 | 0.01 | 0.20 | .845 |
| NLE_N_ x EXT_Full_ | .00 | -0.03 | -0.40 | .686 | 0.00 | 0.03 | 0.34 | .732 |
| NLE_N_ x AV_nocost-cs+_ | .00 | 0.11 | 1.65 | .100 | 0.01 | 0.16 | 2.01 | .046* |
| NLE_N_ x AV_nocost-cs-_ | .00 | 0.02 | 0.23 | .819 | 0.00 | 0.12 | 1.28 | .200 |
| NLE_N_ x GEN | .00 | -0.06 | -0.78 | .437 | 0.01 | -0.20 | -2.43 | .016* |

*Note*. DASS-A, DASS-S, DASS-D = subscales of the Depression Anxiety Stress Scales; GAD = Generalized Anxiety Disorder seven item scale; NLE_N_ = number of negative life events between Part 1 and 2; EXT_Early_ = early extinction index based on expectancy ratings; EXT_Full_ = full extinction index based on expectancy ratings; AV_nocost_csmin_ = proportion avoided CS- trials without cost, AV_nocost_csplus_ = proportion avoided CS+ trials without cost, GEN = generalization index based on expectancy ratings; Part 2 = post-measurement in February; Part 3 = post-measurement in March

^a^ Grey values: compared to the Step 0 model (only intercept); white values: compared to the Step 1 model (indicated in grey).

*** *p* < .001; ** *p* < .01; * *p* < .05

**Table 2**

*Prediction of Changes in Anxiety, Stress, and Depression, in Interaction with the Cumulative Burden of Negative Life Events (Primary Indices)*

|  | **Part 2** | | | | **Part 3** | | | |
| --- | --- | --- | --- | --- | --- | --- | --- | --- |
|  | **∆*R^2^*** | **β** | ***t*** | ***p*** | **∆*R^2^*** | **β** | ***t*** | ***p*** |
| **DASS-A** |  |  |  |  |  |  |  |  |
| NLE_burden_ | .04 | 0.04 | 4.72 | <.001*** | .06 | 0.05 | 5.97 | <.001*** |
| NLE_burden_ x ACQ_csplus_ | .01 | 0.02 | 2.46 | .015* | 0.00 | 0.00 | 0.37 | .709 |
| NLE_burden_ x ACQ_csmin_ | .00 | 0.01 | 1.11 | .268 | 0.01 | 0.02 | 2.06 | .040* |
| NLE_burden_ x EXT_Early_ | .00 | -0.01 | -0.67 | .503 | .00 | 0.00 | -0.41 | .679 |
| NLE_burden_ x EXT_Full_ | .00 | -0.01 | -0.52 | .601 | .00 | 0.00 | -0.39 | .700 |
| NLE_burden_ x AV_nocost-cs+_ | .00 | 0.01 | 0.95 | .341 | .00 | 0.01 | 0.73 | .464 |
| NLE_burden_ x AV_nocost-cs-_ | .00 | 0.00 | 0.41 | .680 | .00 | 0.00 | 0.07 | .948 |
| NLE_burden_ x GEN | .00 | -0.01 | -1.12 | .265 | .01 | -0.02 | -2.08 | .038* |
| **DASS-S** |  |  |  |  |  |  |  |  |
| NLE_burden_ | .07 | 0.07 | 6.43 | <.001*** | .08 | 0.07 | 6.62 | <.001*** |
| NLE_burden_ x ACQ_csplus_ | .02 | 0.03 | 3.26 | .001** | 0.01 | 0.02 | 1.87 | .062 |
| NLE_burden_ x ACQ_csmin_ | .00 | 0.01 | 0.53 | .599 | 0.00 | 0.02 | 1.68 | .094 |
| NLE_burden_ x EXT_Early_ | .00 | 0.00 | -0.06 | .952 | .00 | 0.00 | -0.04 | .970 |
| NLE_burden_ x EXT_Full_ | .00 | -0.01 | -0.50 | .617 | .00 | 0.00 | -0.29 | .774 |
| NLE_burden_ x AV_nocost-cs+_ | .01 | 0.02 | 2.19 | .030* | .00 | 0.02 | 1.33 | .185 |
| NLE_burden_ x AV_nocost-cs-_ | .00 | -0.01 | -0.48 | .632 | .00 | 0.00 | 0.03 | .980 |
| NLE_burden_ x GEN | .01 | -0.02 | -1.64 | .102 | .02 | -0.03 | -2.83 | .005** |
| **DASS-D** |  |  |  |  |  |  |  |  |
| NLE_burden_ | .06 | 0.06 | 5.73 | <.001*** | .05 | 0.06 | 5.30 | <.001*** |
| NLE_burden_ x ACQ_csplus_ | .01 | 0.02 | 2.16 | .031* | 0.00 | 0.01 | 0.93 | .351 |
| NLE_burden_ x ACQ_csmin_ | .00 | 0.00 | -0.47 | .641 | 0.00 | 0.00 | -0.05 | .960 |
| NLE_burden_ x EXT_Early_ | .00 | 0.00 | 0.39 | .698 | .00 | 0.00 | 0.18 | .856 |
| NLE_burden_ x EXT_Full_ | .00 | 0.00 | -0.09 | .924 | .00 | -0.01 | -0.53 | .599 |
| NLE_burden_ x AV_nocost-cs+_ | .00 | 0.02 | 1.39 | .167 | .01 | 0.03 | 2.30 | .022* |
| NLE_burden_ x AV_nocost-cs-_ | .00 | 0.00 | 0.06 | .949 | .00 | 0.02 | 1.19 | .235 |
| NLE_burden_ x GEN | .00 | -0.01 | -0.63 | .529 | .00 | -0.01 | -1.00 | .317 |
| **GAD-7** |  |  |  |  |  |  |  |  |
| NLE_burden_ | .05 | 0.06 | 6.55 | <.001*** | .05 | 0.06 | 5.94 | <.001*** |
| NLE_burden_ x ACQ_csplus_ | .00 | 0.01 | 1.40 | .161 | 0.00 | 0.00 | -0.39 | .698 |
| NLE_burden_ x ACQ_csmin_ | .00 | 0.01 | 0.79 | .431 | 0.01 | 0.02 | 1.93 | .054 |
| NLE_burden_ x EXT_Early_ | .00 | -0.01 | -0.87 | .385 | .00 | 0.00 | -0.26 | .798 |
| NLE_burden_ x EXT_Full_ | .00 | -0.01 | -0.85 | .396 | .00 | 0.00 | -0.37 | .713 |
| NLE_burden_ x AV_nocost-cs+_ | .00 | 0.01 | 0.88 | .382 | .00 | 0.01 | 1.32 | .188 |
| NLE_burden_ x AV_nocost-cs-_ | .00 | 0.00 | -0.36 | .721 | .00 | 0.00 | 0.32 | .748 |
| NLE_burden_ x GEN | .00 | -0.01 | -0.59 | .553 | .01 | -0.03 | -2.54 | .012* |

*Note*. DASS-A, DASS-S, DASS-D = subscales of the Depression Anxiety Stress Scales; GAD = Generalized Anxiety Disorder seven item scale; NLE_burden_ = cumulative burden of negative life events between Part 1 and 2; EXT_Early_ = early extinction index based on expectancy ratings; EXT_Full_ = full extinction index based on expectancy ratings; AV_nocost_csmin_ = proportion avoided CS- trials without cost, AV_nocost_csplus_ = proportion avoided CS+ trials without cost, GEN = generalization index based on expectancy ratings; Part 2 = post-measurement in February; Part 3 = post-measurement in March

^a^ Grey values: compared to the Step 0 model (only intercept); white values: compared to the Step 1 model (indicated in grey).

*** *p* < .001; ** *p* < .01; * *p* < .05

**Table 3**

*Prediction of Changes in Anxiety, Stress, and Depression, in Interaction with the Number of Negative Life Events (Secondary Indices)*

|  | **Part 2** | | | | **Part 3** | | | |
| --- | --- | --- | --- | --- | --- | --- | --- | --- |
|  | **∆*R^2^*** | **β** | ***t*** | ***p*** | **∆*R^2^*** | **β** | ***t*** | ***p*** |
| **DASS-A** |  |  |  |  |  |  |  |  |
| NLE_N_ x ACQ_Dist-csplus_ | .01 | 0.11 | 1.70 | .090 | .01 | 0.15 | 2.19 | .029* |
| NLE_N_ x ACQ_Dist-csmin_ | .00 | 0.07 | 0.98 | .327 | .01 | 0.13 | 1.81 | .070 |
| NLE_N_ x EXT_Dist-Early_ | .00 | -0.08 | -1.34 | .181 | .00 | -0.07 | -1.07 | .287 |
| NLE_N_ x EXT_Dist-Full_ | .00 | -0.06 | -0.95 | .345 | .00 | -0.02 | -0.26 | .794 |
| NLE_N_ x AV_cost-csplus_ | .00 | 0.09 | 1.04 | .298 | .00 | 0.09 | 1.09 | .278 |
| NLE_N_ x GEN_Dist_ | .00 | 0.06 | 0.74 | .459 | .00 | 0.07 | 0.91 | .365 |
| **DASS-S** |  |  |  |  |  |  |  |  |
| NLE_N_ x ACQ_Dist-csplus_ | .00 | 0.10 | 1.32 | .186 | .00 | 0.08 | 1.08 | .281 |
| NLE_N_ x ACQ_Dist-csmin_ | .00 | 0.05 | 0.58 | .561 | .00 | 0.03 | 0.41 | .680 |
| NLE_N_ x EXT_Dist-Early_ | .01 | -0.13 | -1.83 | .069 | .00 | -0.04 | -0.51 | .612 |
| NLE_N_ x EXT_Dist-Full_ | .01 | -0.12 | -1.56 | .120 | .00 | -0.04 | -0.51 | .608 |
| NLE_N_ x AV_cost-csplus_ | .00 | 0.11 | 1.22 | .224 | .00 | 0.10 | 1.04 | .300 |
| NLE_N_ x GEN_Dist_ | .00 | 0.11 | 1.17 | .245 | .00 | -0.04 | -0.39 | .700 |
| **DASS-D** |  |  |  |  |  |  |  |  |
| NLE_N_ x ACQ_csplus_ | .00 | 0.06 | 0.86 | .388 | .01 | 0.18 | 2.25 | .025* |
| NLE_N_ x ACQ_csmin_ | .00 | 0.03 | 0.33 | .741 | .01 | 0.16 | 1.84 | .066 |
| NLE_N_ x EXT_Early_ | .00 | -0.01 | -0.12 | .903 | .00 | -0.07 | -0.96 | .340 |
| NLE_N_ x EXT_Full_ | .00 | -0.02 | -0.24 | .812 | .00 | -0.07 | -0.83 | .406 |
| NLE_N_ x AV_nocost-cs+_ | .01 | 0.15 | 1.66 | .098 | .00 | 0.12 | 1.22 | .224 |
| NLE_N_ x GEN | .00 | 0.08 | 0.85 | .395 | .00 | 0.09 | 0.97 | .334 |
| **GAD-7** |  |  |  |  |  |  |  |  |
| NLE_N_ x ACQ_csplus_ | .00 | 0.05 | 0.83 | .408 | .00 | 0.02 | 0.21 | .834 |
| NLE_N_ x ACQ_csmin_ | .00 | 0.03 | 0.48 | .629 | .00 | 0.01 | 0.11 | .910 |
| NLE_N_ x EXT_Early_ | .00 | -0.03 | -0.48 | .631 | .00 | 0.02 | 0.36 | .722 |
| NLE_N_ x EXT_Full_ | .00 | -0.01 | -0.15 | .881 | .00 | 0.06 | 0.86 | .391 |
| NLE_N_ x AV_nocost-cs+_ | .00 | 0.02 | 0.25 | .799 | .00 | 0.03 | 0.41 | .682 |
| NLE_N_ x GEN | .00 | 0.03 | 0.35 | .727 | .00 | -0.03 | -0.32 | .750 |

*Note*. DASS-A, DASS-S, DASS-D = subscales of the Depression Anxiety Stress Scales; GAD = Generalized Anxiety Disorder seven item scale; NLE_N_ = number of negative life events between Part 1 and 2; EXT_Early_ = early extinction index based on expectancy ratings; EXT_Full_ = full extinction index based on expectancy ratings; AV_nocost_csmin_ = proportion avoided CS- trials without cost, AV_nocost_csplus_ = proportion avoided CS+ trials without cost, GEN = generalization index based on expectancy ratings; Part 2 = post-measurement in February; Part 3 = post-measurement in March

^a^ Grey values: compared to the Step 0 model (only intercept); white values: compared to the Step 1 model (indicated in grey).

*** *p* < .001; ** *p* < .01; * *p* < .05

**Table 4**

*Prediction of Changes in Anxiety, Stress, and Depression, in Interaction with the Cumulative Burden of Negative Life Events (Secondary Indices)*

|  | **Part 2** | | | | **Part 3** | | | |
| --- | --- | --- | --- | --- | --- | --- | --- | --- |
|  | **∆*R^2^*** | **β** | ***t*** | ***p*** | **∆*R^2^*** | **β** | ***t*** | ***p*** |
| **DASS-A** |  |  |  |  |  |  |  |  |
| NLE_burden_ x ACQ_Dist-csplus_ | .00 | 0.01 | 0.87 | .387 | .00 | 0.01 | 1.25 | .211 |
| NLE_burden_ x ACQ_Dist-csmin_ | .00 | 0.01 | 0.80 | .427 | .00 | 0.01 | 1.42 | .158 |
| NLE_burden_ x EXT_Dist-Early_ | .01 | -0.02 | -1.90 | .059 | .01 | -0.02 | -2.56 | .011* |
| NLE_burden_ x EXT_Dist-Full_ | .00 | -0.01 | -1.32 | .189 | .00 | -0.01 | -1.20 | .229 |
| NLE_burden_ x AV_cost-csplus_ | .00 | 0.01 | 1.26 | .210 | .00 | 0.01 | 0.93 | .353 |
| NLE_burden_ x GEN_Dist_ | .00 | 0.01 | 0.81 | .417 | .00 | 0.01 | 1.07 | .287 |
| **DASS-S** |  |  |  |  |  |  |  |  |
| NLE_burden_ x ACQ_Dist-csplus_ | .00 | 0.00 | 0.20 | .839 | .00 | -0.01 | -0.69 | .491 |
| NLE_burden_ x ACQ_Dist-csmin_ | .00 | 0.00 | 0.17 | .867 | .00 | -0.01 | -0.61 | .544 |
| NLE_burden_ x EXT_Dist-Early_ | .01 | -0.03 | -2.43 | .016* | .01 | -0.02 | -1.71 | .089 |
| NLE_burden_ x EXT_Dist-Full_ | .01 | -0.02 | -1.95 | .053 | .00 | -0.01 | -0.93 | .355 |
| NLE_burden_ x AV_cost-csplus_ | .01 | 0.02 | 1.94 | .054 | .00 | 0.01 | 1.01 | .313 |
| NLE_burden_ x GEN_Dist_ | .00 | 0.01 | 1.22 | .222 | .00 | 0.00 | 0.18 | .860 |
| **DASS-D** |  |  |  |  |  |  |  |  |
| NLE_burden_ x ACQ_Dist-csplus_ | .00 | 0.00 | -0.44 | .659 | .00 | 0.01 | 0.95 | .343 |
| NLE_burden_ x ACQ_Dist-csmin_ | .00 | -0.01 | -0.74 | .462 | .00 | 0.01 | 0.94 | .350 |
| NLE_burden_ x EXT_Dist-Early_ | .00 | -0.01 | -0.56 | .574 | .01 | -0.02 | -1.53 | .127 |
| NLE_burden_ x EXT_Dist-Full_ | .00 | -0.01 | -0.50 | .620 | .00 | -0.01 | -1.04 | .299 |
| NLE_burden_ x AV_cost-csplus_ | .01 | 0.02 | 1.48 | .141 | .00 | 0.01 | 0.64 | .521 |
| NLE_burden_ x GEN_Dist_ | .00 | 0.01 | 0.91 | .365 | .00 | 0.02 | 1.28 | .200 |
| **GAD-7** |  |  |  |  |  |  |  |  |
| NLE_burden_ x ACQ_Dist-csplus_ | .00 | -0.01 | -0.84 | .404 | .00 | -0.01 | -1.33 | .185 |
| NLE_burden_ x ACQ_Dist-csmin_ | .00 | -0.01 | -0.62 | .533 | .00 | -0.01 | -0.79 | .431 |
| NLE_burden_ x EXT_Dist-Early_ | .00 | -0.01 | -1.32 | .186 | .00 | -0.01 | -1.43 | .153 |
| NLE_burden_ x EXT_Dist-Full_ | .00 | -0.01 | -0.74 | .457 | .00 | 0.00 | -0.08 | .932 |
| NLE_burden_ x AV_cost-csplus_ | .00 | 0.00 | 0.24 | .807 | .00 | 0.00 | 0.25 | .807 |
| NLE_burden_ x GEN_Dist_ | .00 | 0.00 | -0.05 | .957 | .00 | 0.00 | -0.17 | .869 |

*Note*. DASS-A, DASS-S, DASS-D = subscales of the Depression Anxiety Stress Scales; GAD = Generalized Anxiety Disorder seven item scale; NLE_burden_ = cumulative burden of negative life events between Part 1 and 2; EXT_Early_ = early extinction index based on expectancy ratings; EXT_Full_ = full extinction index based on expectancy ratings; AV_nocost_csmin_ = proportion avoided CS- trials without cost, AV_nocost_csplus_ = proportion avoided CS+ trials without cost, GEN = generalization index based on expectancy ratings; Part 2 = post-measurement in February; Part 3 = post-measurement in March

^a^ Grey values: compared to the Step 0 model (only intercept); white values: compared to the Step 1 model (indicated in grey).

*** *p* < .001; ** *p* < .01; * *p* < .05
